# Asperinsuterpenes A–C from the Fungus *Aspergillus insuetus* BTBU20220155

**DOI:** 10.3390/jof10090611

**Published:** 2024-08-27

**Authors:** Xinjun Zhang, Fuhang Song, Jiahui Han, Long Wang, Linlin Ma, Xiuli Xu

**Affiliations:** 1 Key Laboratory of Forest Ecology in Tibet Plateau (Ministry of Education), Institute of Tibet Plateau Ecology, Tibet Agriculture & Animal Husbandry University, Nyingchi 860000, China; zxjun_126abc@126.com; 2 Key Laboratory of Geriatric Nutrition and Health, Ministry of Education of China, School of Light Industry Science and Engineering, Beijing Technology and Business University, Beijing 100048, China; 3 Key Laboratory of Polar Geology and Marine Mineral Resources, Ministry of Education, School of Ocean Sciences, China University of Geosciences, Beijing 100083, China; 15632779760@163.com (J.H.);; 4 State Key Lab of Mycology, Institute of Microbiology, Chinese Academy of Sciences, Beijing 100101, China; wl_dgk@im.ac.cn; 5 Institute for Biomedicine and Glycomics, School of Environment and Science, Griffith University, Brisbane, QLD 4111, Australia

**Keywords:** *Aspergillus insuetus*, Tibet, spiromeroterpenoid, *Candida albicans*

## Abstract

Three new meroterpenoids, asperinsuterpenes A–C (**1**–**3**), and eight previously reported natural products, namely asnovolin I (**4**), (2′*E*,4′*E*,6′*E*)-6-(1′-carboxyocta-2′,4′,6′-triene)-9-hydroxydrim-7-ene-11,12-olide (**5**), (2′*E*,4′*E*,6′*E*)-6-(1′-carboxyocta-2′,4′,6′-triene)-11,12-epoxy-9,11-dihydroxydrim-7-ene (**6**), cinereain (**7**), carnequinazolines A and B (**8** and **9**), carnemycin B (**10**), and stromemycin (**11**) were isolated from the fungus *Aspergillus insuetus,* strain BTBU20220155. The structures of the compounds were determined based on spectroscopic techniques, including 1D and 2D NMR, HRESIMS, and ECD experiments. The in vitro antimicrobial evaluation revealed that compounds **5** and **11** exhibited inhibitory activity against *Candida albicans*, with minimum inhibitory concentration (MIC) values of 12.5 and 25 μg/mL, respectively. These findings suggest that *A. insuetus* is a promising source of bioactive natural products with potential applications in antifungal therapy.

## 1. Introduction

Fungi have long been recognized as a vital source of structurally and biologically significant natural products, including penicillin, cyclosporin A, and lovastatin. Among fungi, *Aspergillus* species, which thrive in diverse environments such as soil, leaves, water, marine settings, and decaying food, are particularly noteworthy. Numerous bioactive compounds, such as heterocyclic alkaloids, sesquiterpenoids, polyketides, and pyrones, have been identified from *Aspergillus* fungi [1,2,3,4].

*Aspergillus insuetus*, belonging to the *Aspergillus* section Usti, was first described in 1929 [5]. This filamentous fungus is found in various environments, such as soil, foods, indoor air, and samples collected from marine environments [5,6]. Chemical investigations of *A. insuetus* have revealed numerous compounds with diverse bioactivates, including drimane sesquiterpenes [6,7,8,9], phenol derivatives [10], meroterpenoids, and depsipeptides [11].

As part of our ongoing screening of active natural products from fungi, we isolated a strain of *A. insuetus* BTBU20220155 from a soil sample collected from the Sejila Mountain, Tibet, China. To diversify the new chemical entries from *A. insuetus*, a systematic chemical investigation of *A. insuetus* BTBU20220155 was performed, which led to the identification of three new compounds **1**–**3**, along with eight previously reported compounds from other fungal sources, including drimane sesquiterpene esters, asnovolin I (**4**), (2′*E*,4′*E*,6′*E*)-6-(1′-carboxyocta-2′,4′,6′-triene)-9-hydroxydrim-7-ene-11,12-olide (**5**), (2′*E*,4′*E*,6′*E*)-6-(1′-carboxyocta-2′,4′,6′-triene)-11,12-epoxy-9,11-dihydroxydrim-7-ene (**6**) [12], cinereain (**7**) [13], carnequinazolines A and B (**8** and **9**), carnemycin B (**10**), [14], and stromemycin (**11**) [15,16]. The isolated compounds were evaluated for their in vitro antifungal and antibacterial activities against *Candida albicans*, *Staphylococcus aureus*, and *Escherichia coli*, respectively. Herein, we present the fermentation, compound isolation, structural characterization, and bioactivity evaluation of the isolated compounds.

## 2. Materials and Methods

### 2.1. Molecular Identification

The fungus *A. insuetus* BTBU20220155 was isolated from a soil sample collected from Sejila Mountain, Tibet, China. This strain was cultured on a potato dextrose agar plate for 7 days at 28 °C. The genomic DNA of *A. insuetus* BTBU20220155 was extracted using a Fungi Genomic DNA Kit (Solarbio, Beijing, China), and the internal transcribed spacer (ITS) region was amplified using the conventional primer pair of ITS5 (5′-GGAAGTAAAAGTCGTAACAAGG-3′) and ITS4 (5′-TCCTCCGCTTATTGATATGC-3′). Then, the sequence of PCR products was determined by Beijing Qingke Biotechnology Co., Ltd. (Beijing, China). The ITS sequence of strain BTBU20220155 was compared against the GenBank database using the BLAST program. The Sequence similarity was determined using multiple sequence alignment with CLUSTAL W [17]. The strain has been deposited at Beijing Technology and Business University, Beijing, China, with the accession number BTBU20220155.

### 2.2. General Experimental Procedure

The optical rotations ([α]_D_) were determined using an Anton Paar MCP 200 Modular Circular Polarimeter (Graz, Austria). The circular dichroism (CD) spectra were recorded on an Applied Photophysics Chirascan spectropolarimeter (Surrey, UK). The NMR spectra were obtained on a Bruker Avance 500 spectrometer (Fällanden, Switzerland), with residual solvent peaks as references (Acetone-*d*_6_: *δ*_H_ 2.05, *δ*_C_ 28.4). High-resolution ESIMS measurements were obtained on an Accurate-Mass-Q-TOF LC/MS 6520 instrument (Santa Clara, CA, USA). HPLC was performed using an Agilent 1200 Series separation module with a diode array detector (Santa Clara, CA, USA), 1260 Series fraction collector, and Agilent XDB-C8, RX-C8, and XDB-C18 columns (250 × 9.4 mm, 5 µm).

### 2.3. Fungal Materials, Cultivation, Fermentation, and Isolation

*A. insuetus* BTBU20220155 was cultured on potato dextrose agar (Solarbio, Beijing, China) at 28 °C for 7 days. Small sections (approximately 1 cm^2^) were inoculated into 1 L conical flasks (× 15), each containing 150 mL of distilled water and 200 g of rice (Taiyuxiang, COFCO, Beijing, China). The flasks were incubated stationary at 28 °C for 24 days. The cultured medium with fungi was extracted using a mixture of EtOAc:MeOH (80:20). The whole culture and extraction procedure was repeated three times, and the combined extracts were concentrated to dryness in vacuum, producing a thick liquid, which is usually referred to as a dark residue. The residue was partitioned between EtOAc and H_2_O. Then, the EtOAc was removed in vacuo to yield a dark residue (5.63 g).

The EtOAc fraction was subjected to vacuum silica gel chromatography (50 × 80 mm column, TLC H silica), eluting with a stepwise gradient of 50–100% hexane/CH_2_Cl_2_ and then 100–10% CH_2_Cl_2_/MeOH, resulting in 10 fractions. Fraction M was further purified on a Sephadex LH-20 column (600 × 30 mm), eluting with an isocratic elution with CH_2_Cl_2_:MeOH (2:1), yielding six subfractions (F1–F6). Subfraction F1 was further fractionated by HPLC (Agilent Eclipse XDB-C8, 250 × 9.4 mm, 5 μm column, 3.0 mL/min, isocratic 45% MeCN/H_2_O with isocratic 0.01% TFA modifier) to yield compound **1** (t_R_ 11.836 min, 11.5 mg). Subfraction F2 was separated by HPLC (Agilent Eclipse XDB-C8, 250 × 9.4 mm, 5 μm column, 3.0 mL/min, isocratic 45% MeCN/H_2_O with isocratic 0.01% TFA modifier) to yield F2-1, F2-2, F2-3 and F2-4, respectively. F2-3 was further fractionated by HPLC (Agilent Eclipse XDB-C8, 250 × 9.4 mm, 5 μm column, 3.0 mL/min, isocratic 50% MeCN/H_2_O) to give compound **2** (t_R_ 11.453 min, 10.7 mg), compound **4** (t_R_ 11.453 min, 26.2 mg) and compound **3** (t_R_ 11.618 min, 8.1 mg). Subfraction F4 was subjected to HPLC (Agilent Zorbax RX-C8, 250 × 9.4 mm, 5 μm column, 3.0 mL/min, gradient elution from 30 to 100% MeCN/H_2_O over 30 min) to yield compound **8** (t_R_ 10.795 min, 5.3 mg), compound **7** (t_R_ 10.957 min, 7.6 mg), compound **9** (t_R_ 11.313 min, 3.4 mg), compound **5** (t_R_ 13.083 min, 5.3 mg), and compound **6** (t_R_ 13.847 min, 24.8 mg), respectively. Fraction Q was separated on a Sephadex LH-20 column (600 × 30 mm) using an isocratic elution with CH_2_Cl_2_:MeOH (2:1), to give five subfractions (F1–F5). Subfraction F4 was subjected to column chromatographic separation by HPLC (Agilent Eclipse XDB-C18, 250 × 9.4 mm, 5 μm column, 3.0 mL/min, gradient elution from 45–70% MeCN/H_2_O over 25 min with isocratic 0.01% TFA modifier) to yield compound **10** (t_R_ 12.470 min, 59.5 mg) and compound **11** (t_R_ 10.183 min, 10.5 mg).

Asperinsuterpene A (**1**): Colorless amorphous powder; ${\left[\alpha \right]}_{D}^{22}$ + 154 (MeOH, 0.1); (+)-HRESIMS *m*/*z* 441.2273 [M + H]^+^ (calcd. for C_26_H_32_O_6_ 441.2272); ^1^H and ^13^C NMR data: See Table 1.

Asperinsuterpene B (**2**): Colorless amorphous powder; ${\left[\alpha \right]}_{D}^{22}$ + 55 (MeOH, 0.1); (+)-HRESIMS *m*/*z* 497.2518 [M + Na]^+^ (calcd. for C_27_H_38_O_7_Na 497.2510); ^1^H and ^13^C NMR data: See Table 1.

Asperinsuterpene C (**3**): Colorless amorphous powder; ${\left[\alpha \right]}_{D}^{22}$ + 3 (MeOH, 0.1); (+)-HRESIMS *m*/*z* 445.2583 [M + H]^+^ (calcd. for C_26_H_37_O_6_ 445.2585); ^1^H and ^13^C NMR data: See Table 2.

### 2.4. ECD Calculation Methods

The ECD calculation was carried out using previously reported methods [18]. The conformers were submitted for geometric optimization at the level of CAM-B3LYP/6-31g(d) with the Gaussian 16 software package using the DFT method [19]. Excited state calculations of their low-energy conformations were performed using the TDDFT (time-dependent density functional theory) at the same level.

### 2.5. Antimicrobial Assay

The antimicrobial evaluations were carried out based on the Antimicrobial Susceptibility Testing Standards outlined by the Clinical and Laboratory Standards Institute (CLSI) against *S. aureus* (ATCC 6538), *E. coli* (ATCC 11775), and *C. albicans* (ATCC 10231), using a 96-well plate microdilution method. Briefly, the bacterial strains were inoculated on LB agar plates and cultured overnight at 37 °C. *C. albicans* was inoculated on sabouraud dextrose agar and cultured at 28 °C for 24 h. Single colonies were picked into Mueller-Hinton Broth for bacterial suspension and into RPMI 1640 media for fungal suspension and cultured overnight. The suspensions were adjusted to approximately 10^6^ and 10^4^ CFU/mL for bacteria and fungus, respectively. Each compound (2 μL), serially diluted 2-fold in DMSO, was added into each row of a 96-well microplate containing 78 μL of bacterial or fungal suspension per well. Vancomycin and ciprofloxacin (Solarbio, Beijing, China) were used as the positive controls for *S. aureus* and *E. coli*, respectively, while amphotericin B (Solarbio, Beijing, China) served as the positive control for *C. albicans*. DMSO was used as the negative control. The plates were cultured aerobically at 37 °C for 18 h for bacteria and at 35 °C for 24 h for *C. albicans*. The Minimum Inhibitory Concentration (MIC) was defined as the lowest concentration of the compound at which no visible microbial growth was observed based on the standard set by the Clinical and Laboratory Standards Institute (CLSI).

## 3. Results and Discussion

### 3.1. Phylogenetic Analysis

**Figure 1:**
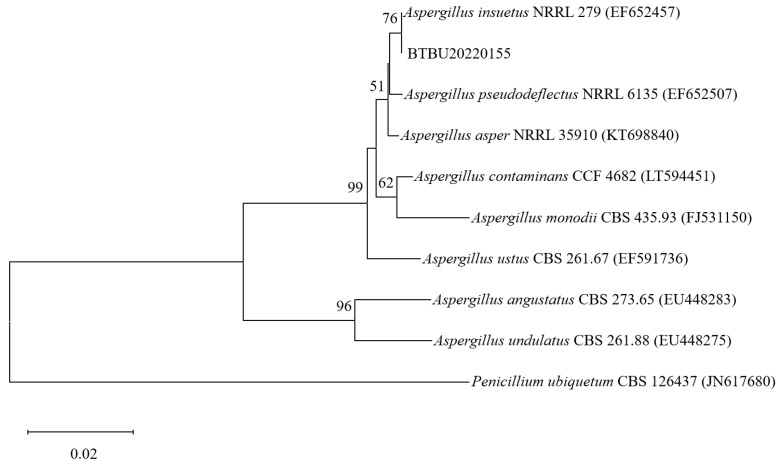
Maximum likelihood analysis based on ITS sequence. Bootstrap values ≥ 50% are indicated at the nodes. The tree was rooted to *Penicillium. ubiquetum* CBS 126437.

The phylogenetic tree of BTBU20220155 constructed according to the ITS rDNA sequences is shown in Figure 1. The strain BTBU20220155 exhibited the highest identity (99.81%) with *A. insuetus* NRRL 279. Phylogenetically, the strain BTBU20220155 was identified as *A. insuetus*.

### 3.2. Structure Elucidation

**Figure 2:**
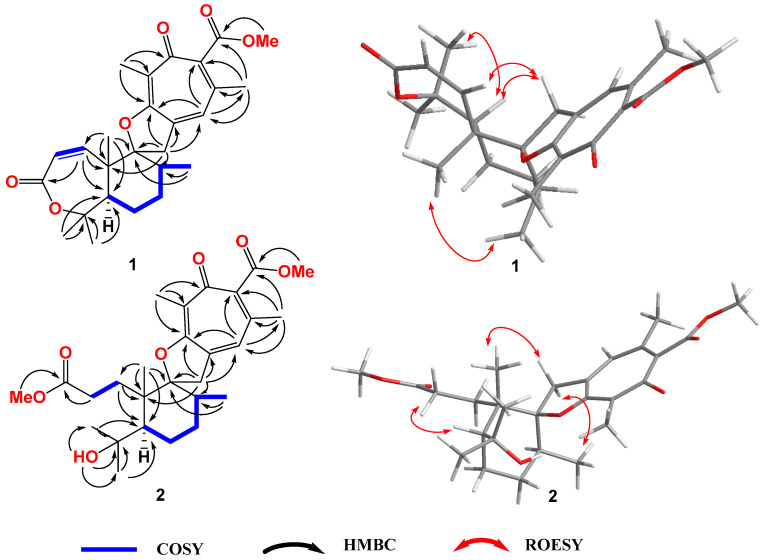
Key COSY, HMBC, and ROESY correlations in compounds **1** and **2**.

Asperinsuterpene A (compound **1)** was obtained as a colorless, amorphous powder. The molecular formula of compound **1** was determined to be C_26_H_32_O_6_ by HRESIMS, with *m*/*z* 441.2273 [M + H]^+^ (calcd. for C_26_H_33_O_6_ 441.2272, Δmmu + 0.1) (Figure S1), accounting for eleven degrees of unsaturation. The ^1^H, ^13^C (Table 1) and HSQC NMR spectra (Figures S2–S4 in Supplementary Materials) revealed the presence of three carbonyl carbons, five olefinic quaternary carbons, three sp^2^-hybridized methines, three sp^3^-hybridized methylenes, two sp^3^-hybridized methines, three sp^3^-hybridized quaternary carbons (two oxygenated), and seven methyl groups (including one methyl doublet, one methoxy group and five methyl singlets). These functional groups accounted for seven of the eleven degrees of unsaturation, which indicated that compound **1** was tetracyclic. Ring A was established as a seven-membered unsaturated lactone based on the ^1^H–^1^H COSY correlations (Figure 2) between H-1 (*δ*_H_ 6.32, d, *J* = 13.0 Hz) and H-2 (*δ*_H_ 5.90, d, *J* = 13.0 Hz), and the HMBC correlations from H-1 to C-3 (*δ*_C_ 166.0), C-5 (*δ*_C_ 51.5), and C-10 (*δ*_C_ 51.5), and from H_3_-25 (*δ*_H_ 1.41, s) and H_3_-26 (*δ*_H_ 1.50, s) to C-4 (*δ*_C_ 84.3) and C-5. The mutual correlations between C-25 and the C-26 methyl singlets further supported this structure. The ^1^H–^1^H COSY correlations from H-5 (*δ*_H_ 2.16, dd, *J* = 8.5, 6.5 Hz) to H_3_-24 (*δ*_H_ 1.22, d, *J* = 8.5 Hz) through the H_2_-6 (*δ*_H_ 1.83, m), H_2_-7 (*δ*_H_ 1.91, m; 1.73, m), and H_2_-8 (*δ*_H_ 2.12, m) correlations, the HMBC correlations from H_3_-23 (*δ*_H_ 1.57, s) to C-5, C-9 (*δ*_C_ 92.8) and C-10, as well as those from H_3_-24 to C-8 (*δ*_C_ 39.0) and C-9 revealed the presence of a cyclohexane ring B. Ring C was established to be a dihydrofuran moiety based on the HMBC correlations (Figure 2) from H-11α to C-9, C-12 (*δ*_C_ 141.8), and C-18 (*δ*_C_ 163.5), as well as from H-11β to C-12 and C-18. The tropone ring D was indicated by the HMBC correlations from H-13 (*δ*_H_ 6.87, brs) to C-15 (*δ*_C_ 143.6), C-18 (*δ*_C_ 163.5) and C-19 (*δ*_C_ 23.9), from H_3_-19 (*δ*_H_ 2.14, s) to C-13 (*δ*_C_ 131.2), C-14 (*δ*_C_ 139.8) and C-15, and from H_3_-22 (*δ*_H_ 2.13, s) to C-16 (*δ*_C_ 181.3), C-17 (*δ*_C_ 127.8) and C-18. These HMBC correlations indicated the presence of a pentasubstituted 2,4,6 cycloheptatriene-1-one ring.

**Figure 3:**
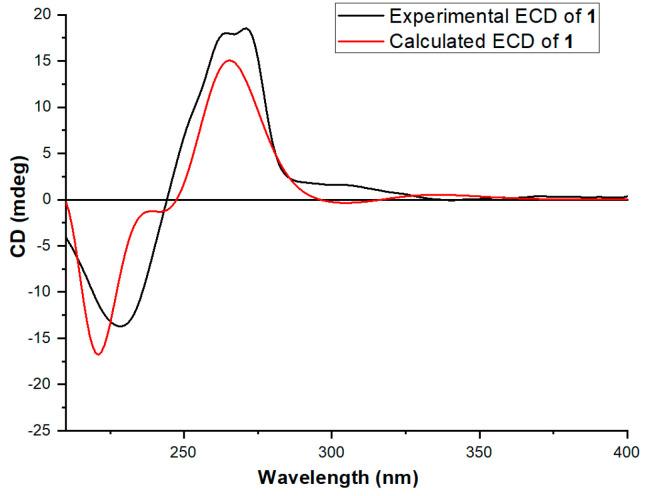
The experimental and calculated ECD spectra of compound **1**.

The carbomethoxy group was defined based on the chemical shifts in C-20 (*δ*_C_ 169.2), H_3_-21 (*δ*_H_ 3.78, s) and C-21 (*δ*_C_ 52.0), and the HMBC correlation from H_3_-21 to C-20. The weak HMBC correlation from H_3_-19 to C-20 revealed the connection of C-15 and C-20. Rings A and B were combined according to the HMBC correlations from H_3_-23 to C-1, C-5, C-9, and C-10. The linkage of rings B and C through a spiro carbon (C-9) was revealed by the HMBC correlations from H-11α to C-8, C-9, and C-10. Additionally, the HMBC correlations from H-11α and H-11β to C-12, C-13, and C-18 indicated the combination of rings C and D. The relative stereochemistry of compound **1** was determined by ROESY spectra. The ROESY correlation between H-11α (*δ*_H_ 3.31) and H-1 (*δ*_H_6.32) revealed the *cis*-form of C-11 and C-1. The ROESY correlation between H_3_-23 and H_3_-24 revealed that these two methyl groups were on the same side of ring B. The ROESY correlations (Figure 2 and Figure S7) between H-5 and H_2_-11 suggested that they are on the same side of ring B. The *cis*-form of H-5 and H_3_-25 was determined by the ROESY correlation from H-5 to H_3_-25. Therefore, the planar structure of compound **1** was determined. By comparing the calculated and experimental ECD spectra (Figure 3), the absolute configuration of compound **1** was determined to be 5*R*, 8*S*, 9*S*, 10*S*.

Asperinsuterpene B (compound **2**) was obtained as a colorless, amorphous powder. The molecular formula of compound **2** was determined as C_27_H_38_O_7_ by HRESIMS with an *m*/*z* of 497.2518 [M + Na]^+^ (calcd. for C_27_H_38_O_7_Na 497.2510, Δmmu + 0.8) (Figure S8), accounting for nine degrees of unsaturation. A comparison of the ^1^H and ^13^C NMR data (Table 1, Figures S9 and S10) with those of compound **1** showed very similar chemical shifts, except for a saturated sidechain (*δ*_H_ 2.07, 2.00, m/*δ*_H_ 35.2; *δ*_H_ 2.44/*δ*_C_ 30.3), an additional methoxy group (*δ*_H_ 3.59, s/*δ*_C_ 51.6), as well as an extra hydroxyl group at *δ*_H_ 3.45. These data revealed that compound **2** is a ring A hydrolyzed methyl ester derivative of compound **1**. Detailed HMBC correlations (Figure 2 and Figure S13), particularly from 4-OH (*δ*_H_ 3.45) to C-4 (*δ*_C_ 74.8), C-25 (*δ*_C_ 28.9), and C-26 (*δ*_C_ 34.5), and from H_3_-27 to C-3 (*δ*_C_ 174.6), supported the proposed assignment of the planar structure of compound **2**. The ROESY correlations between H-5 (*δ*_H_ 1.73, m) and H_2_-2 (*δ*_H_ 2.44, m), and between H-11α (*δ*_H_ 3.62) and H_3_-23 (*δ*_H_ 1.25), indicated that they are on the same side of the cyclohexane ring, respectively. The upward equatorial methyl group of C-24 was confirmed by the ROESY correlations (Figure 2 and Figure S14) from H-11β (*δ*_H_ 3.20) to H_3_-24 (*δ*_H_ 0.88). The optical rotation of compound **2** showed the same orientation as that of compound **1**, so the absolute configuration of compound **2** was determined as 5*R*, 8*S*, 9*S*, 10*S*.

**Figure 4:**
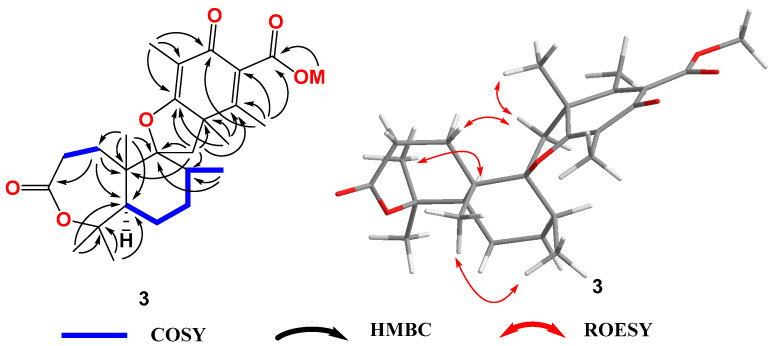
Key COSY, HMBC, and ROESY correlations in compound **3**.

**Figure 5:**
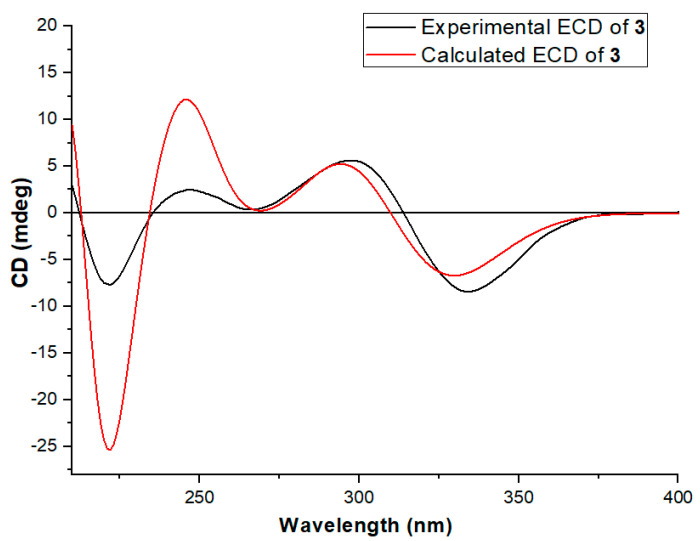
The calculated and experimental ECD spectra of compound **3**.

Asperinsuterpene C (compound **3**) was obtained as a colorless, amorphous powder. The molecular formula of compound **3** was determined to be C_26_H_36_O_6_ by HRESIMS, with an *m*/*z* of 445.2583 [M + H]^+^ (calcd. for C_26_H_37_O_6_ 445.2585, Δmmu—0.2) (Figure S15 in Supplementary Materials), accounting for nine degrees of unsaturation. A comparison of the ^1^H and ^13^C NMR data (Table 2, Figures S16 and S17) with those of compound **1** revealed several similarities, but also significant differences. Compound **3** contained four olefinic carbons instead of eight. Two olefinic carbons in compound **1** were replaced by one methyl singlet and one more sp^3^-hybridized quaternary carbon in compound **3**. The other two olefinic carbons in compound **1** were replaced by two sp^3^ methylenes. This suggested the absence of a tropone ring in compound **3**. In the HMBC spectrum (Figure 4 and Figure S20), the correlations from H_3_-18 (*δ*_H_ 1.60, s) to C-12 (*δ*_C_ 48.8), C-13 (*δ*_C_ 156.1) and C-17 (*δ*_C_ 179.8), from H_3_-19 (*δ*_H_ 2.05, s) to C-12, C-13 and C-15 (*δ*_C_ 184.2), as well as from H_3_-22 (*δ*_H_ 1.75, s) to C-15, C-16 (*δ*_C_ 108.8) and C-17 indicated the presence of a 2,5-cyclohexadiene-1-one ring in compound **3**. The HMBC correlations from H_3_-19 to C-20 (*δ*_C_ 168.1) and from H_3_-21 (*δ*_H_ 3.74, s) to C-20 revealed the attachment of a carbomethoxy group to C-14. The relative stereochemistry of compound **3** was determined by a detailed analysis of the ROESY data (Figure 4 and Figure S21). The correlations from H-11α (*δ*_H_ 2.35) to H_3_-18 (*δ*_H_ 1.60s) and H_2_-1 (*δ*_H_ 1.91 and 1.82) indicated the *cis*-form of H-11α to H_3_-18. The ROESY correlation between H_3_-23 (*δ*_H_ 1.39) and H_3_-24 (*δ*_H_ 0.94) indicated that they were upward axial methyl groups. The correlation from H_3_-25 (*δ*_H_ 1.45, s) to H-5 (*δ*_H_ 2.07, m) defined the positions of H_3_-25 and H_3_-26 (*δ*_H_ 1.42, s). The calculated ECD spectrum (Figure 5) of the 5*R*, 8*S*, 9*S*, 10*S*, 12*R* configuration for compound **3** was consistent with the experimental data. Therefore, the structure of compound **3** was determined.

By comparing the NMR data (Tables S1–S7), the known compounds were identified as asnovolin I (**4**) [20], (2′*E*,4′*E*,6′*E*)-6-(1′-carboxyocta-2′,4′,6′-triene)-9-hydroxydrim-7-ene-11,12-olide (**5**), (2′*E*,4′*E*,6′*E*)-6-(1′-carboxyocta-2′,4′,6′-triene)-11,12-epoxy-9,11-dihydroxydrim-7-ene (**6**) [12], cinereain (**7**) [13], carnequinazolines A and B (**8** and **9**), carnemycin B (**10**) [14], and stromemycin (**11**) [16].

### 3.3. Antimicrobial Activities of the Isolated Compounds

The antimicrobial activities of the isolated compounds were tested against *C. albicans*, *S. aureus*, and *E. col*. None of the tested compounds displayed antibacterial activity against *S. aureus and E. coli* (the MICs for vancomycin and ciprofloxacin were 1 and 0.03125 μg/mL, respectively.). However, compounds **5** and **11** showed antifungal activity against *C. albicans*, with MIC values of 12.5 and 25 μg/mL, respectively. Compound **5** has been identified from *Aspergillus* spp. isolated from marine and desert environments. It showed cytotoxic activity against L5178Y, HeLa, and PC12 cell lines [21], and endothelin receptor-binding inhibitory activity [12]. But compound **5** did not inhibit the growth of phytopathogenic *Fusarium oxysporum*, *F. graminearum*, *Colletotrichum musae*, and *C. gloeosporioides* [6]. Compound **11**, inhibiting the growth of *Ralstonia solanacearum* and *Bacillus subtilis*, has been identified in *Emericella variecolor* [16] and *A. ustus* [22,23]. Although these compounds showed weaker antifungal activity compared to amphotericin B (MIC = 0.5 μg/mL), this is the first report of antifungal activity for compounds **5** and **11**.

## 4. Conclusions

**Figure 6:**
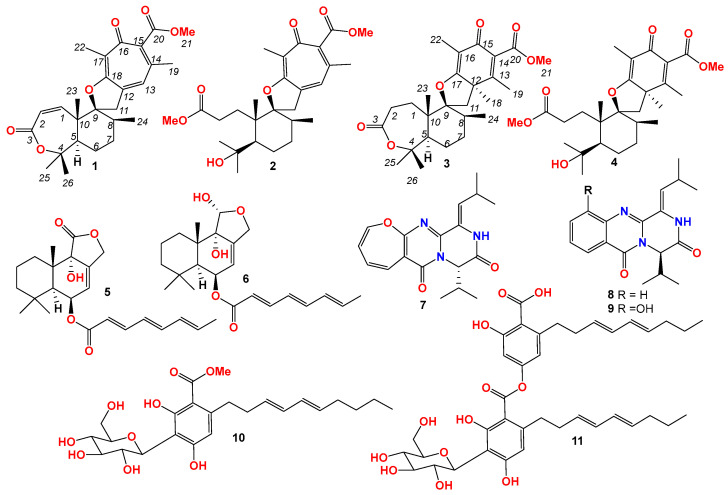
Structures of compounds **1**–**11**.

Fungi have been proven to be important sources for the discovery of new structures and bioactive compounds. In our continuous screening program, which aims to discover novel compounds from fungi, a strain of *A. insuetus* was isolated from a soil sample collected from Sejila Mountain, Tibet, China. To date, only six papers have reported the chemical investigation of *A. insuetus* strains. To explore the chemical diversity of *A. insuetus*, we performed a scale-up fermentation and chemical investigation of *A. insuetus* BTBU20220155. This led to the characterization of three new meroterpenoids, asperinsuterpenes A–C (compounds **1**–**3**), along with eight previously reported natural products (compounds **4**–**11**, Figure 6).

The planar structures and relative configurations of the new compounds were confirmed by detailed analyses of the HRESIMS, 1D and 2D NMR spectroscopic data. Furthermore, the absolute configurations were determined via a comparison of the calculated and experimental electronic circular dichroism (ECD) spectra. Compounds **1** and **2** possess the carbon skeleton of tropolactone analogues, which have been found in a marine-derived fungus isolated from a sponge sample collected 40 feet from Manele Bay, Lanai, Hawaii [24]. However, the ring system in compounds **1** and **2**, originally cyclohexane-tetrahydropyran, was rearranged into a spiro-cyclohexane-dihydrofuran. The carbon skeleton of compound **3** is similar to that of the asnovolins identified from *A. novofumigatus* [25].

Compounds **5** and **11** demonstrated antifungal activity against *C. albicans.* The antifungal activities of these compounds underscore the potential use of *A. insuetus* as a source of bioactive compounds with therapeutic applications. The specific mechanisms through which these compounds exert their antifungal effects could be the subject of further studies, including detailed biochemical assays and molecular docking studies to identify potential targets within fungal cells.

These findings contribute to the growing body of research on fungal metabolites and their potential uses in developing new antifungal therapies, particularly in an era in which resistance to existing antifungal drugs is becoming increasingly problematic.

## Figures and Tables

**Table 1:** ^1^H (500 MHz) and ^13^C (125 MHz) NMR data of **1** and **2** (Acetone-*d*_6_).

Position	1		2	
	*δ*_C_	*δ*_H,_ (*J* in Hz)	*δ*_C_	*δ*_H_, (*J* in Hz)
1	148.5, CH	6.32, d (13.0)	35.2, CH_2_	2.07, m; 2.00, m
2	121.7, CH	5.90, d (13.0)	30.3, CH_2_	2.44, m
3	166.0, C		174.6, C	
4	84.3, CH		74.8, C	
5	51.5, CH	2.16, dd (8.5, 6.5)	50.7, CH	1.73, m
6α	21.2, CH_2_	1.83, m	23.1, CH_2_	1.88, m
6β				1.66, m
7α	29.9, CH_2_	1.91, m	29.6, CH_2_	1.70, m
7β		1.73, m		1.60, m
8	39.0, CH	2.12, m	37.4, CH	2.03, m
9	92.8, C		96.7, C	
10	51.5, C		46.4, C	
11α	44.5, CH_2_	3.31, dd (18.0, 2.0)	40.4, CH_2_	3.62, dd (19.0, 2.0)
11β		3.26, dd (18.0, 1.5)		3.20, dd (19.0, 2.0)
12	141.9, C		143.1, C	
13	131.2, CH	6.87, brs	130.0, CH	6.91, s
14	139.8, C		139.6, C	
15	143.6, C		143.4, C	
16	181.3, C		181.3, C	
17	127.8, C		126.3, C	
18	163.5, C		165.1, C	
19	23.9, CH_3_	2.14, s	23.9, CH_3_	2.15, s
20	169.2, C		169.2, C	
21	52.0, OCH_3_	3.78, s	51.9, OCH_3_	3.76, s
22	13.3, CH_3_	2.13, s	13.7, CH_3_	2.09, s
23	18.0, CH_3_	1.57, s	18.7, CH_3_	1.25, s
24	16.9, CH_3_	1.22, d (7.5)	16.7, CH_3_	0.88, d (7.0)
25α	32.1, CH_3_	1.41, s	28.9, CH_3_	1.31, s
26β	25.6, CH_3_	1.50, s	34.5, CH_3_	1.34, s
27			51.6, CH_3_	3.59, s
4-OH				3.45, s

**Table 2:** ^1^H (500 MHz) and ^13^C (125 MHz) NMR data of **3** ((Acetone-*d*_6_)).

Position	3	
	*δ*_C_	*δ*_H,_ (*J* in Hz)
1a	33.2, CH_2_	1.91, m
1b		1.82, m
2a	32.8, CH_2_	2.74, m
2b		2.68, ddd (15.0, 7.5, 4.0)
3	173.9, C	
4	85.1, C	
5	50.0, CH	2.07, m
6α	22.3, CH_2_	1.84, m
6β		1.55, m,
7α	29.9, CH_2_	1.83, m
7β		1.51, m
8	38.6, CH	1.85, m
9	100.7, C	
10	44.4, C	
11α	37.5, CH_2_	2.35, d (13.5)
11β		2.23, d (13.5)
12	48.8, C	
13	156.1, C	
14	132.8, C	
15	184.2, C	
16	108.8, C	
17	179.8, C	
18	35.0, CH_3_	1.60, s
19	16.8, CH_3_	2.05, s
20	168.1, C	
21	52.0, OCH_3_	3.74, s
22	8.1, CH_3_	1.75, s
23	17.5, CH_3_	1.39, s
24	16.6, CH_3_	0.94, d (6.5)
25	32.3, CH_3_	1.45, s
26	25.1, CH_3_	1.42, s

## Data Availability

The original contributions presented in the study are included in the article/Supplementary Materials, further inquiries can be directed to the corresponding author.

## Supplementary Materials

The following supporting information can be downloaded at: https://www.mdpi.com/article/10.3390/jof10090611/s1, Figures S1–S7: HRESIMS, ^1^H, ^13^C, HSQC, ^1^H–^1^H COSY, HMBC, and ROESY spectra for compound **1**; Figures S8–S14: HRESIMS, ^1^H, ^13^C, HSQC, ^1^H–^1^H COSY, HMBC, and ROESY spectra for compound **2**; Figures S15–S21: HRESIMS, ^1^H, ^13^C, HSQC, ^1^H–^1^H COSY, HMBC, and ROESY spectra for compound **3**. Tables S1–S7: ^13^C NMR data for compounds **3**–**11**.
