# Supplementary material for: Asperinsuterpenes A–C from the Fungus Aspergillus insuetus BTBU20220155
Source: J Fungi (Basel). 2024 Aug 27;10(9):611. doi: 10.3390/jof10090611 (PMC11433199; doi:10.3390/jof10090611)
Supplement: Supplementary file 1 [file jof-10-00611-s001.zip › jof-3120826-supplementary.pdf]

# SUPPLEMENTARY MATERIAL

# Asperinsuterpenes A – C from the fungus *Aspergillus insuetus* BTBU20220155

**Xinjun Zhang [1], Fuhang Song [2*], Jiahui Han [3], Long Wang [4], Linlin Ma [5] and Xiuli Xu [3]**

[1] Institute of Tibet Plateau Ecology, Key Laboratory of Forest Ecology in Tibet Plateau (Ministry of Education), Tibet Agriculture & Animal Husbandry University, Nyingchi, 860000, China
[2] Key Laboratory of Geriatric Nutrition and Health, Ministry of Education of China, School of Light Industry Science and Engineering, Beijing Technology and Business University, Beijing 100048, China
[3] Key Laboratory of Polar Geology and Marine Mineral Resources (China University of Geosciences, Beijing), Ministry of Education; School of Ocean Sciences, China University of Geosciences, Beijing 100083, China
[4] State Key Lab of Mycology, Institute of Microbiology, Chinese Academy of Sciences, Beijing 100101, China
[5] Institute for Biomedicine and Glycomics, School of Environment and Science, Griffith University, Brisbane, QLD 4111, Australia
* Correspondence: songfuhang@btbu.edu.cn

## Table of Contents

**Figure S1.** HRESIMS spectrum for **1** .......... 4
**Figure S2.** $^{1}$H NMR spectrum (500 MHz, Acetone-$d_6$) of **1** .......... 4
**Figure S3.** $^{13}$C NMR spectrum (125 MHz, Acetone-$d_6$) of **1** .......... 5
**Figure S4.** HSQC spectrum (500 MHz, Acetone-$d_6$) of **1** .......... 5
**Figure S5.** $^{1}$H -$^{1}$H COSY spectrum (500 MHz, Acetone-$d_6$) of **1** .......... 6
**Figure S6.** HMBC spectrum (500 MHz, Acetone-$d_6$) of **1** .......... 6
**Figure S7.** ROESY spectrum (500 MHz, Acetone-$d_6$) of **1** .......... 7
**Figure S8.** HRESIMS spectrum for **2** .......... 7
**Figure S9.** $^{1}$H NMR spectrum (500 MHz, Acetone-$d_6$) of **2** .......... 8
**Figure S10.** $^{13}$C NMR spectrum (125 MHz, Acetone-$d_6$) of **2** .......... 8
**Figure S11.** HSQC spectrum (500 MHz, Acetone-$d_6$) of **2** .......... 9
**Figure S12.** $^{1}$H -$^{1}$H COSY spectrum (500MHz, Acetone-$d_6$) of **2** .......... 9
**Figure S13.** HMBC spectrum (500MHz, Acetone-$d_6$) of **2** .......... 10
**Figure S14.** ROESY spectrum (500MHz, Acetone-$d_6$) of **2** .......... 10
**Figure S15.** HRESIMS spectrum for **3** .......... 11
**Figure S16.** $^{1}$H NMR spectrum (500 MHz, Acetone-$d_6$) of **3** .......... 11
**Figure S17.** $^{13}$C NMR spectrum (125 MHz, Acetone-$d_6$) of **3** .......... 12
**Figure S18.** HSQC spectrum (500 MHz, Acetone-$d_6$) of **3** .......... 12

**Figure S19.** $^{1}H$ -$^{1}H$ COSY spectrum (500MHz, Acetone-$d_6$) of **3** ............................ 13
**Figure S20.** HMBC spectrum (500 MHz, Acetone-$d_6$) of **3** ...................................... 13
**Figure S21.** ROESY spectrum (500 MHz, Acetone-$d_6$) of **3** ..................................... 14
**Table S1.** $^{13}C$ NMR data for compound **4**.................................................................. 15
**Table S2.** $^{13}C$ NMR data for compound **5**.................................................................. 16
**Table S3.** $^{13}C$ NMR data for compound **6**.................................................................. 17
**Table S4.** $^{13}C$ NMR data for compound **7**.................................................................. 18
**Table S5.** $^{13}C$ NMR data for compound **8**.................................................................. 19
**Table S6.** $^{13}C$ NMR data for compound **9**.................................................................. 20
**Table S7.** $^{13}C$ NMR data for compound **10**................................................................ 21
**Table S8.** $^{13}C$ NMR data for compound **11**................................................................ 22
**Preparation of tested compounds and positive controls** ....................................... 23
**Antibacterial assay** ......................................................................................... 23
**Antifungal assay** ........................................................................................... 23

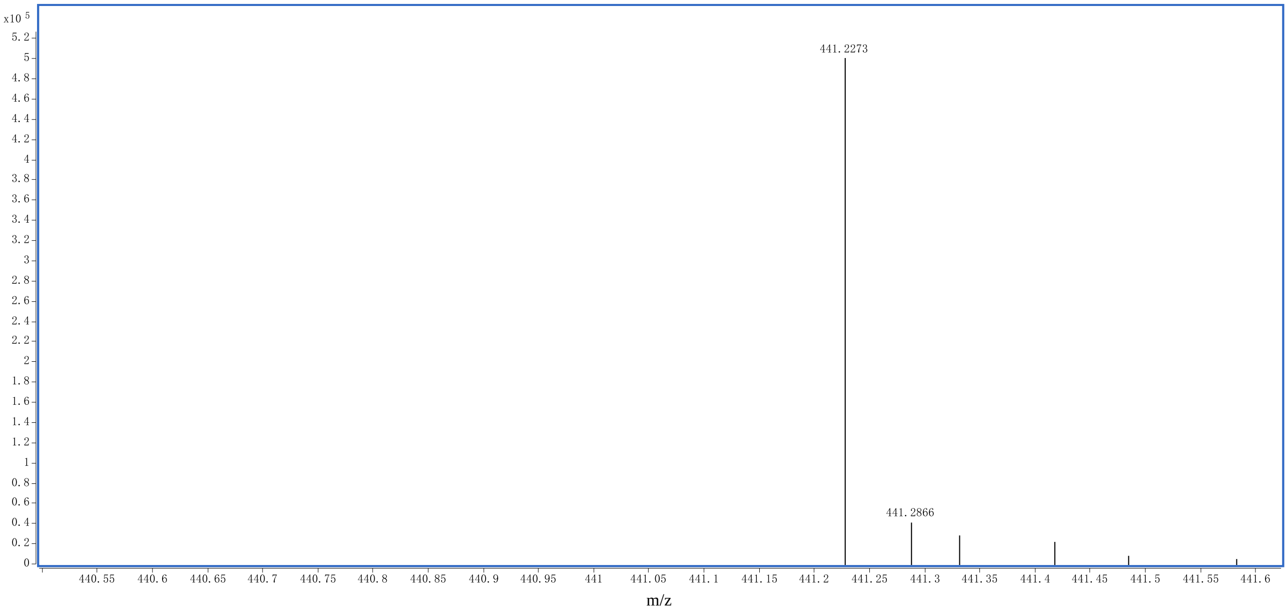


**Figure S1.** HRESIMS spectrum for **1**

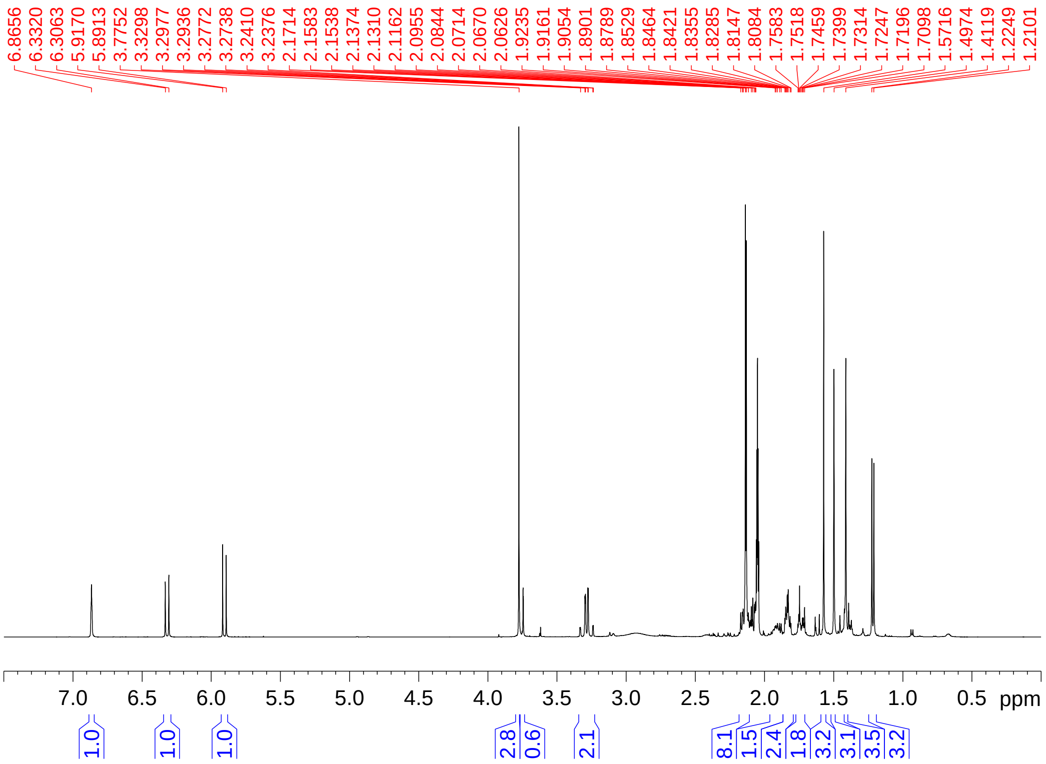


**Figure S2.** $^{1}$H NMR spectrum (500 MHz, Acetone-$d_6$) of **1**

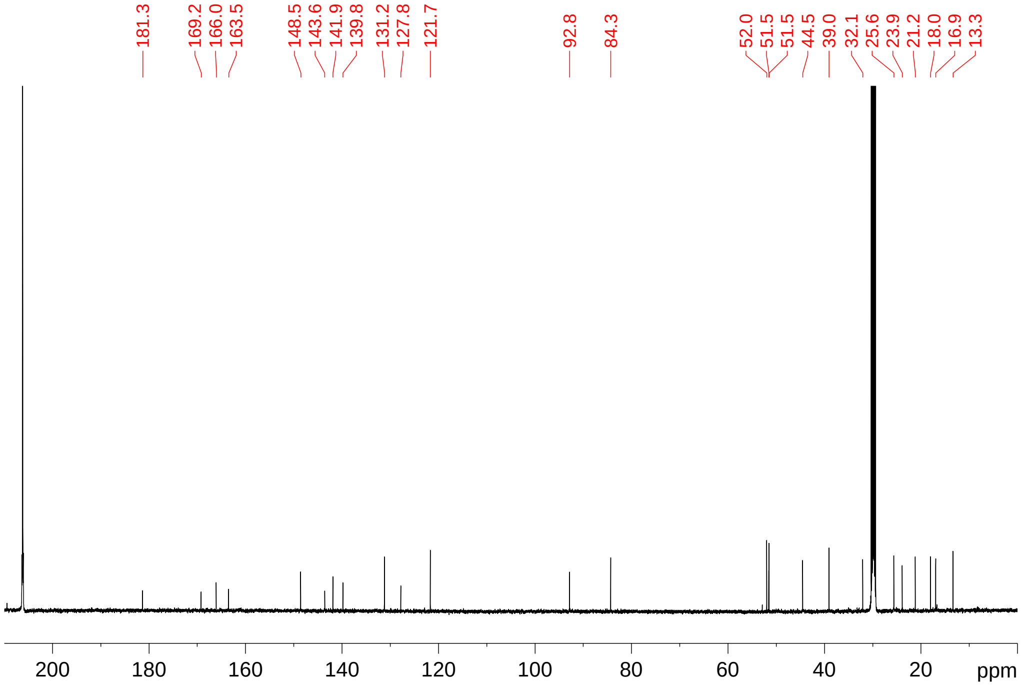


**Figure S3.** $^{13}$C NMR spectrum (125 MHz, Acetone-$d_6$) of **1**

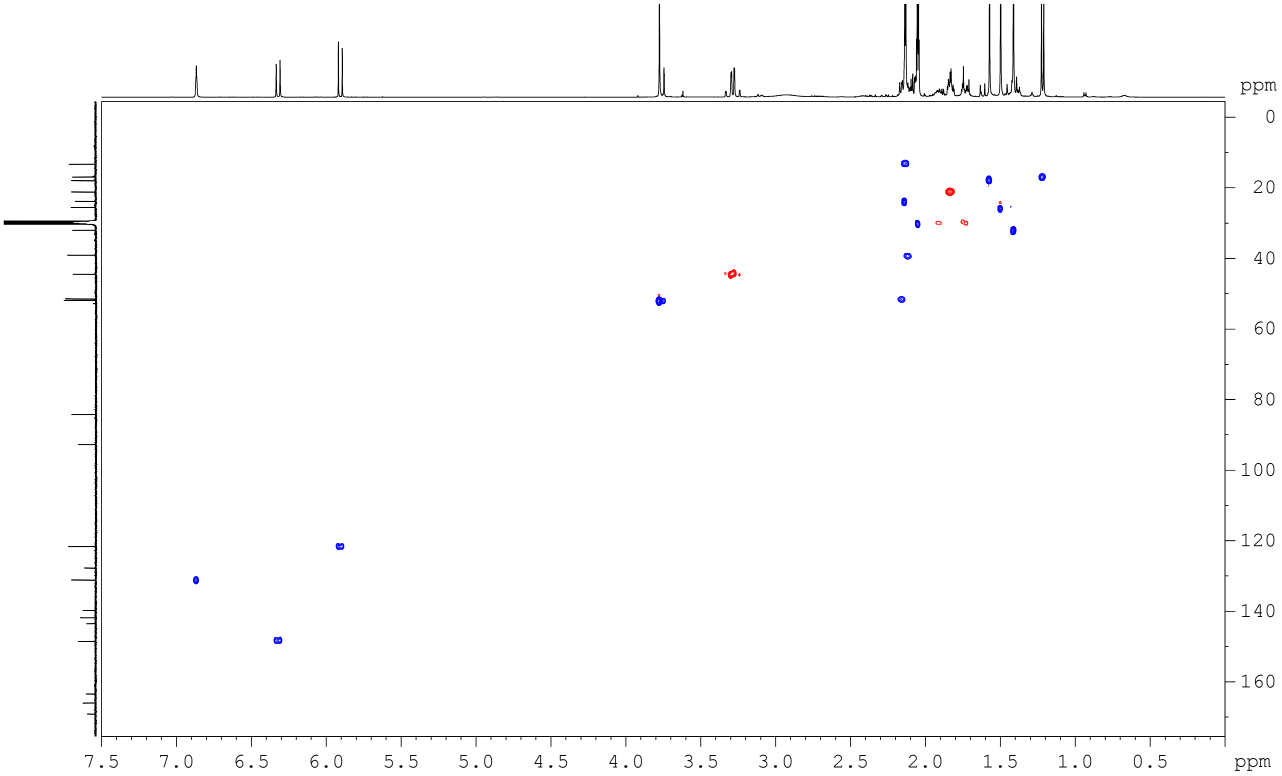


**Figure S4.** HSQC spectrum (500 MHz, Acetone-$d_6$) of **1**

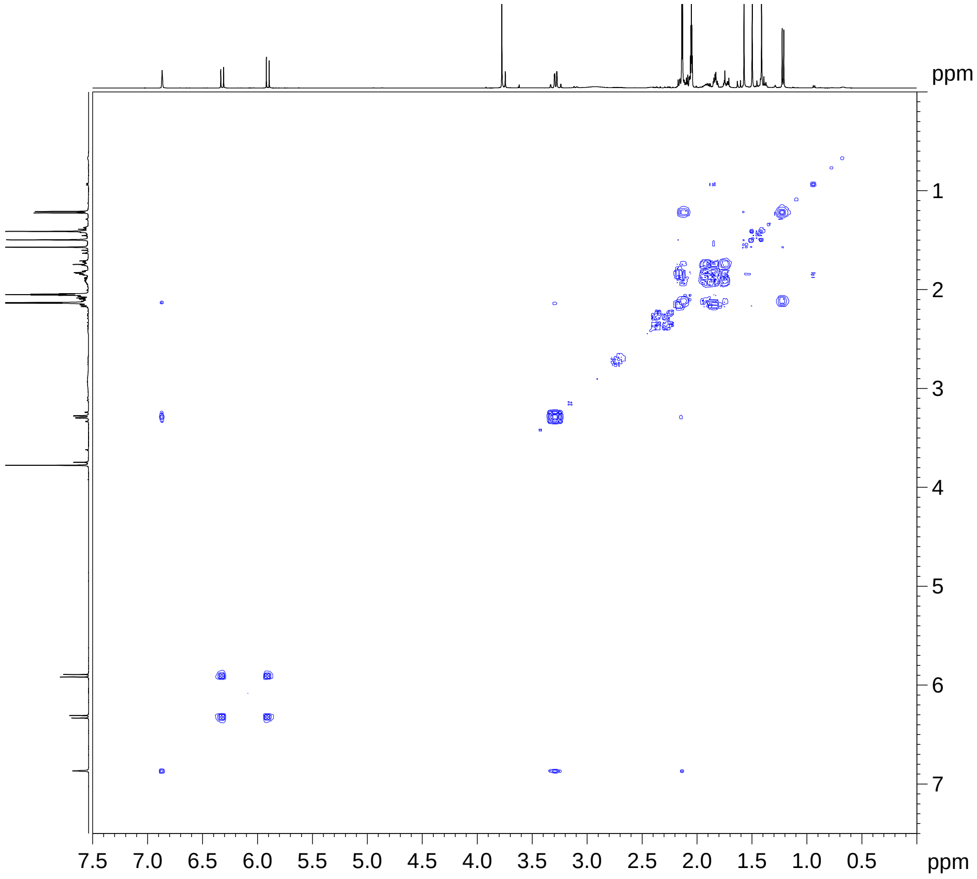

**Figure S5.** $^{1}H$ -$^{1}H$ COSY spectrum (500 MHz, Acetone-$d_6$) of **1**

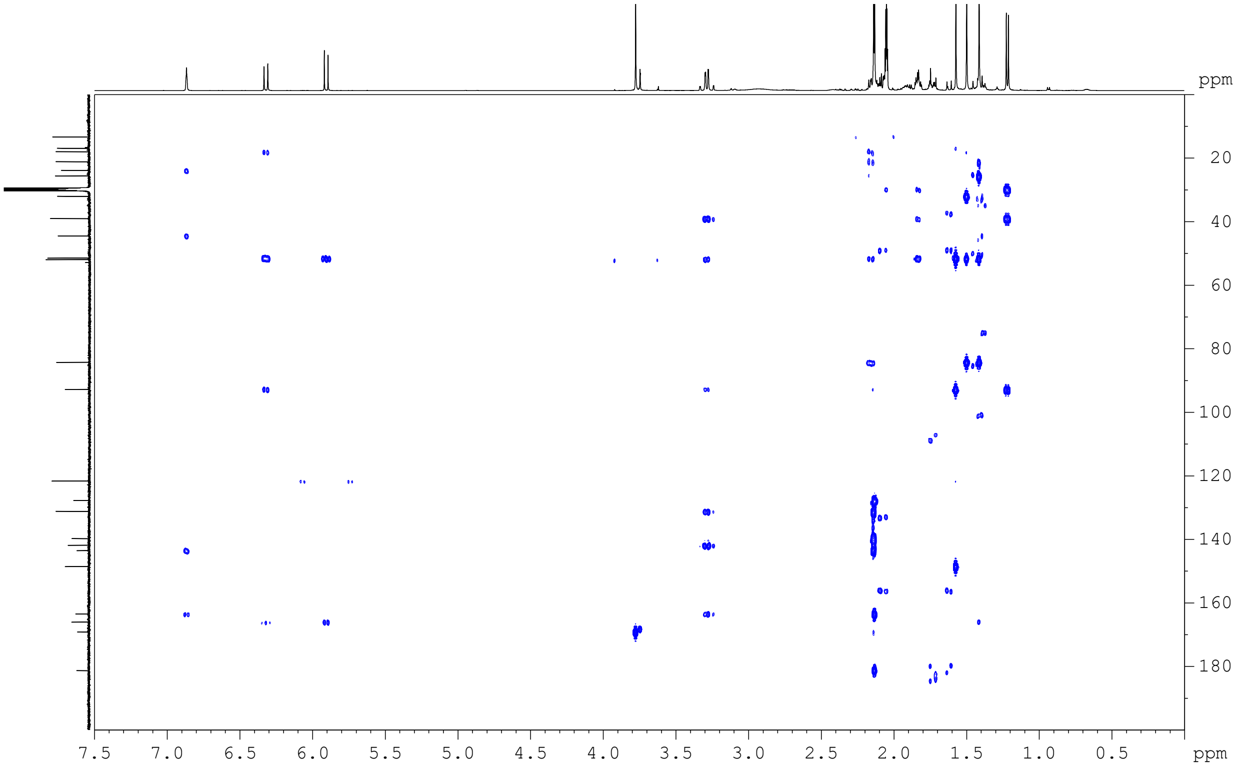


**Figure S6.** HMBC spectrum (500 MHz, Acetone-$d_6$) of **1**

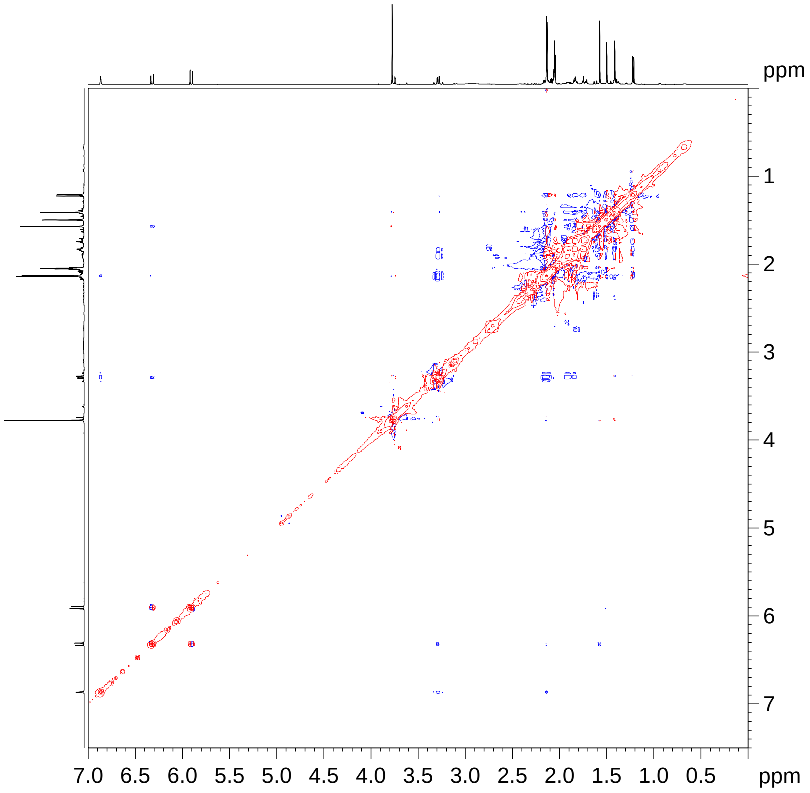


**Figure S7.** ROESY spectrum (500 MHz, Acetone-$d_6$) of **1**

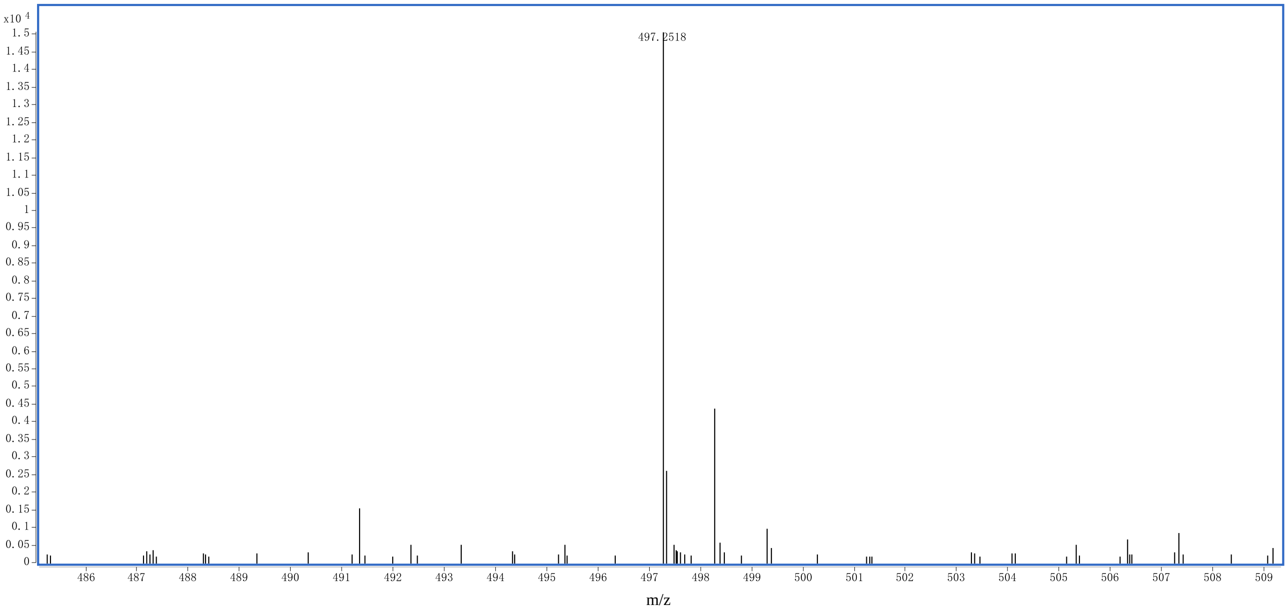


**Figure S8.** HRESIMS spectrum of **2**

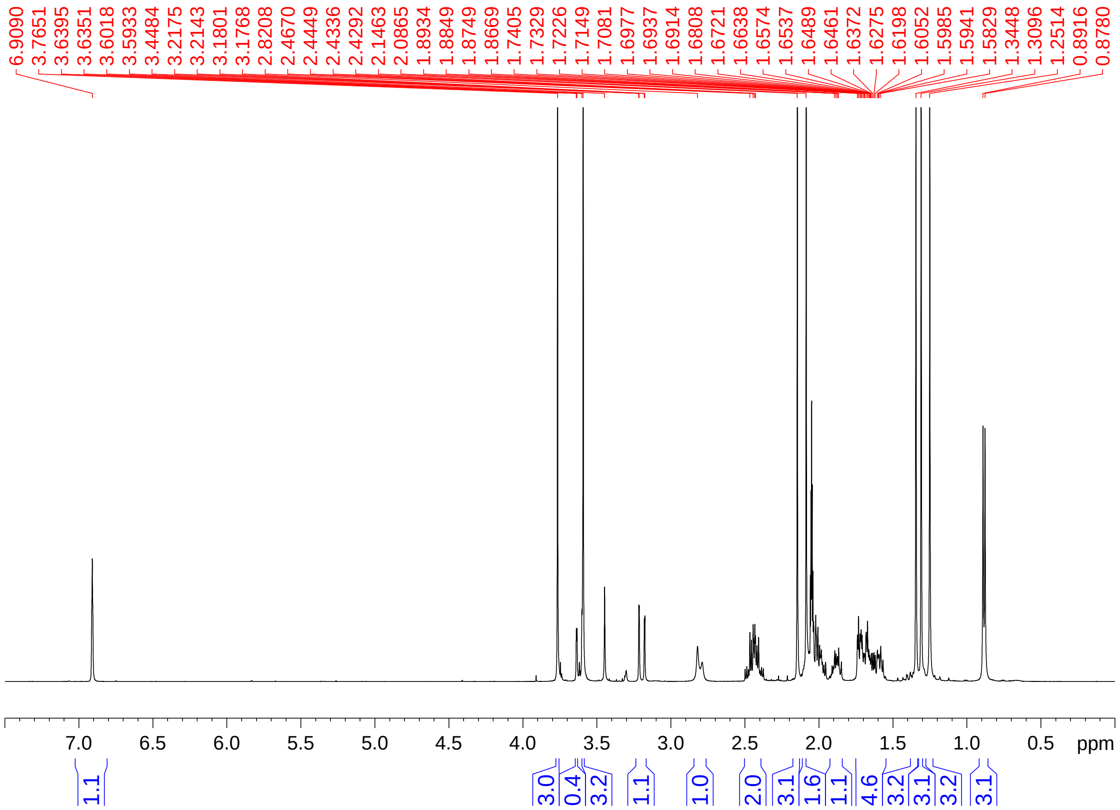


**Figure S9.** $^{1}$H NMR spectrum (500 MHz, Acetone-$d_6$) of **2**

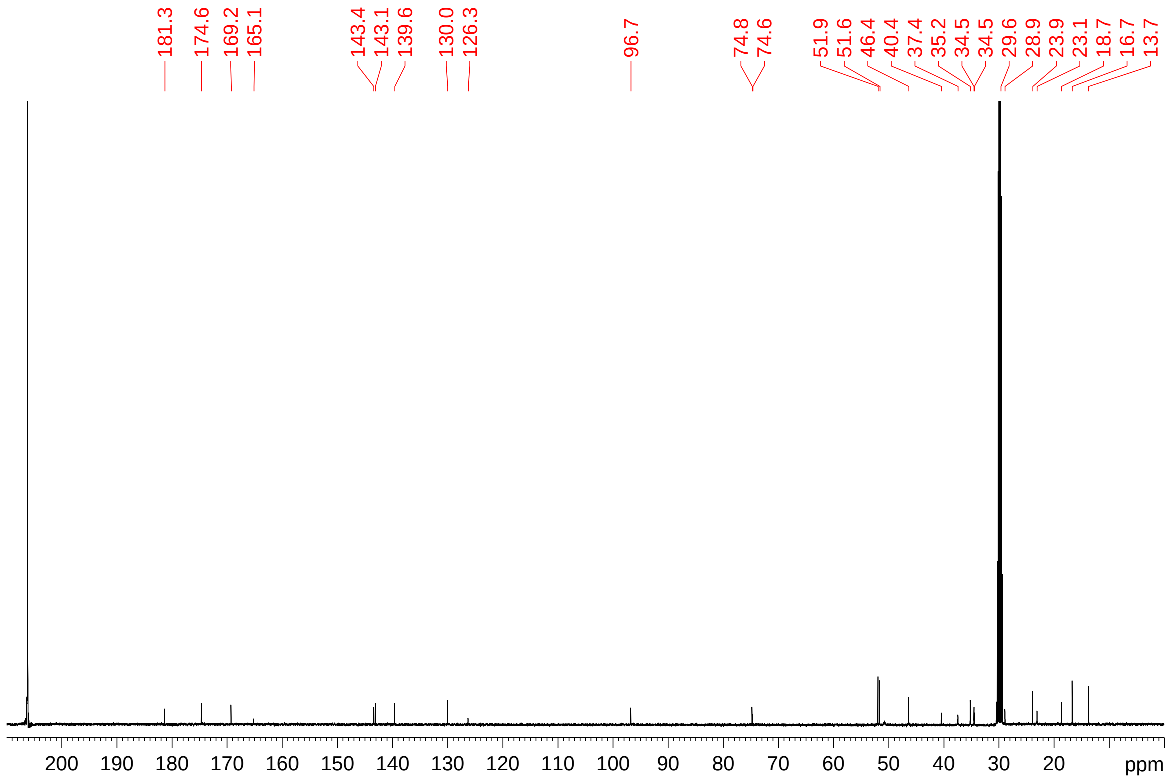


**Figure S10.** $^{13}$C NMR spectrum (125 MHz, Acetone-$d_6$) of **2**

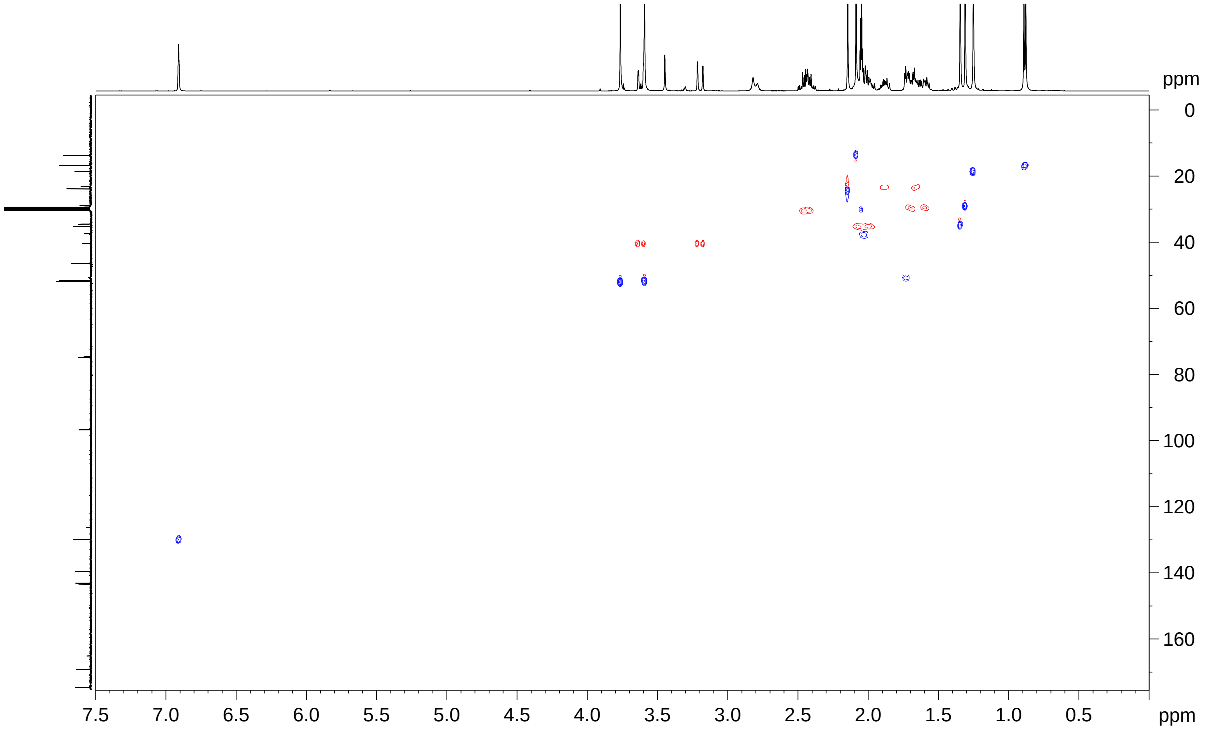


**Figure S11.** HSQC spectrum (500 MHz, Acetone-$d_6$) of **2**

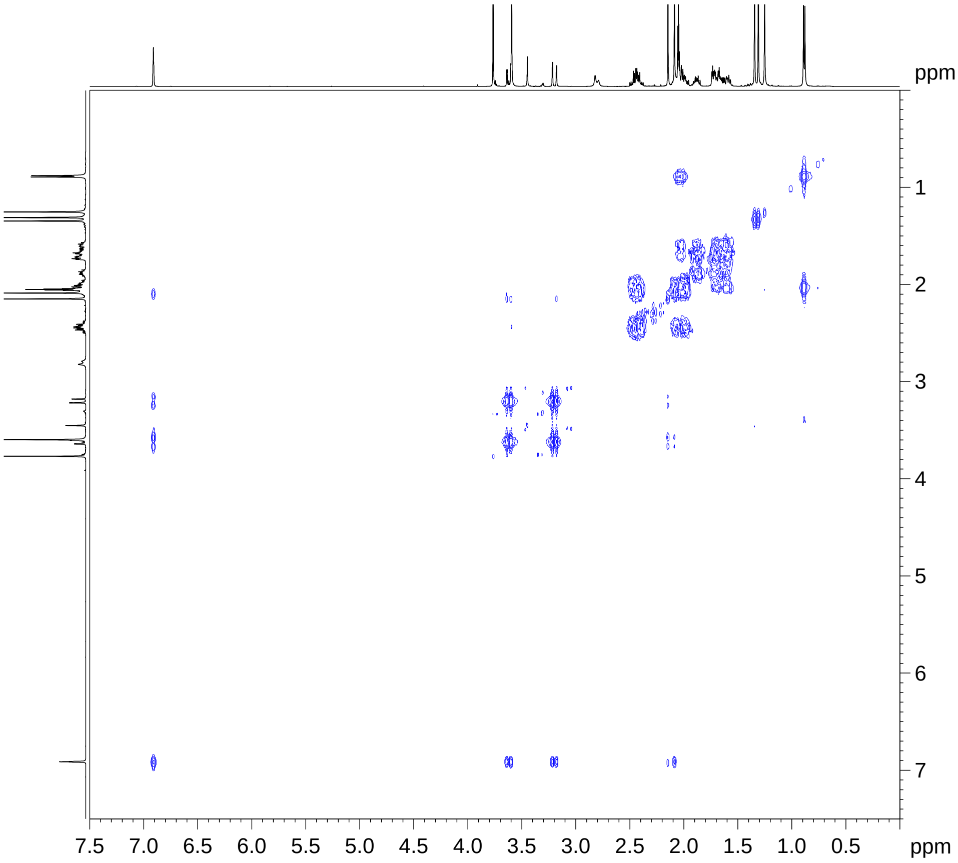


**Figure S12.** $^{1}$H -$^{1}$H COSY spectrum (500MHz, Acetone-$d_6$) of **2**

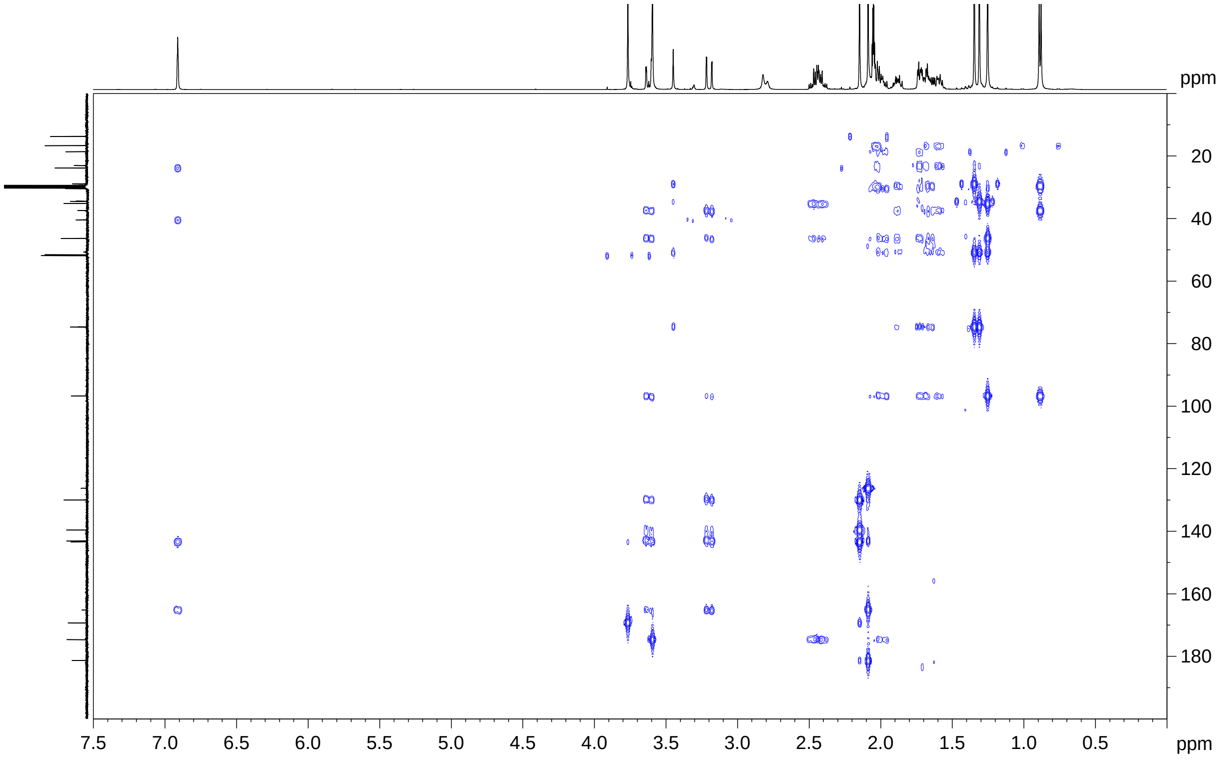

**Figure S13.** HMBC spectrum (500 MHz, Acetone-$d_6$) of **2**

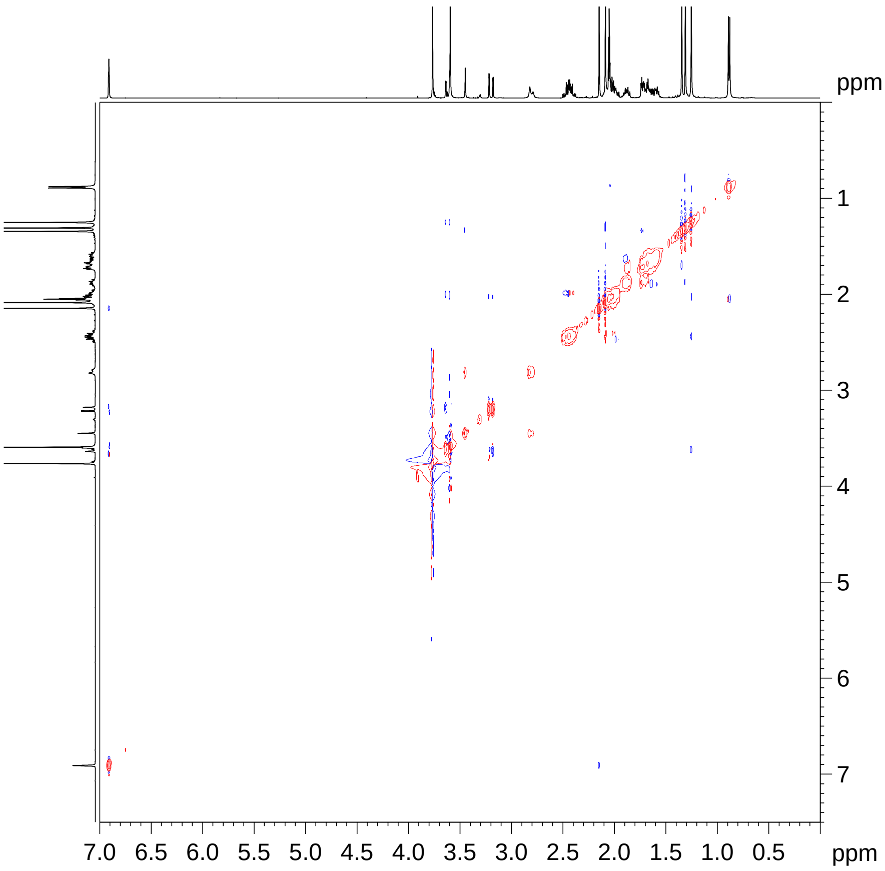


**Figure S14.** ROESY spectrum (500 MHz, Acetone-$d_6$) of **2**

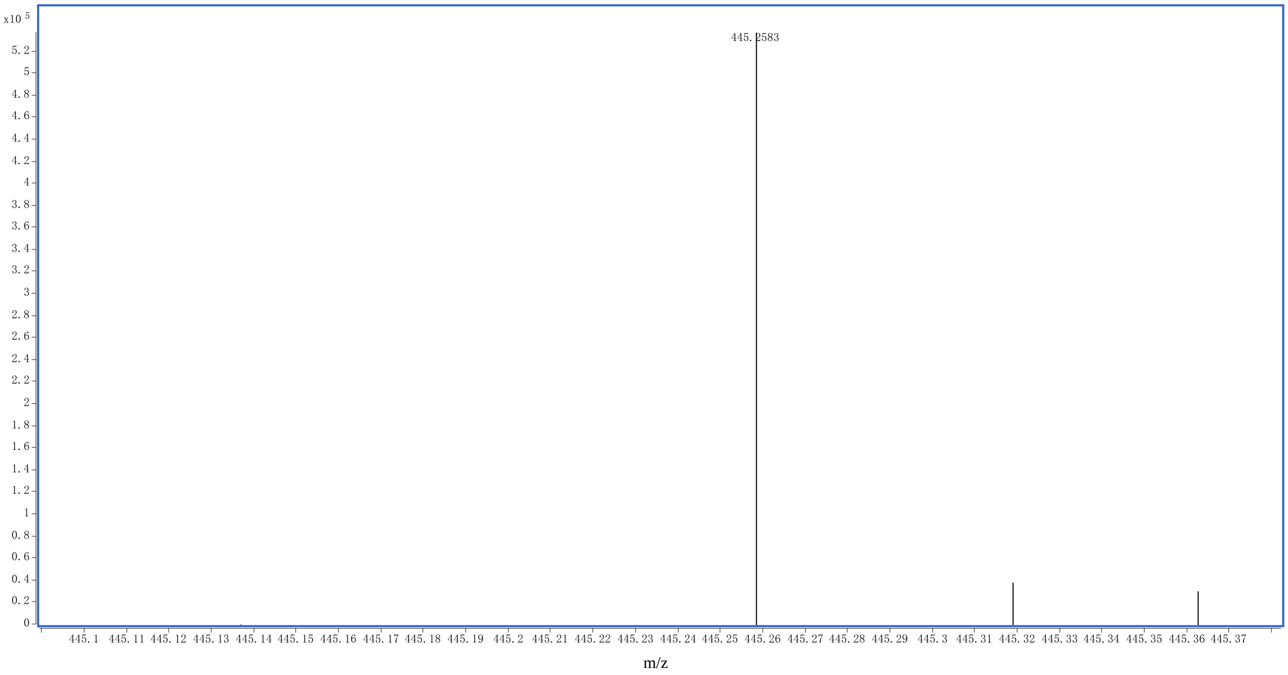


**Figure S15.** HRESIMS spectrum of **3**

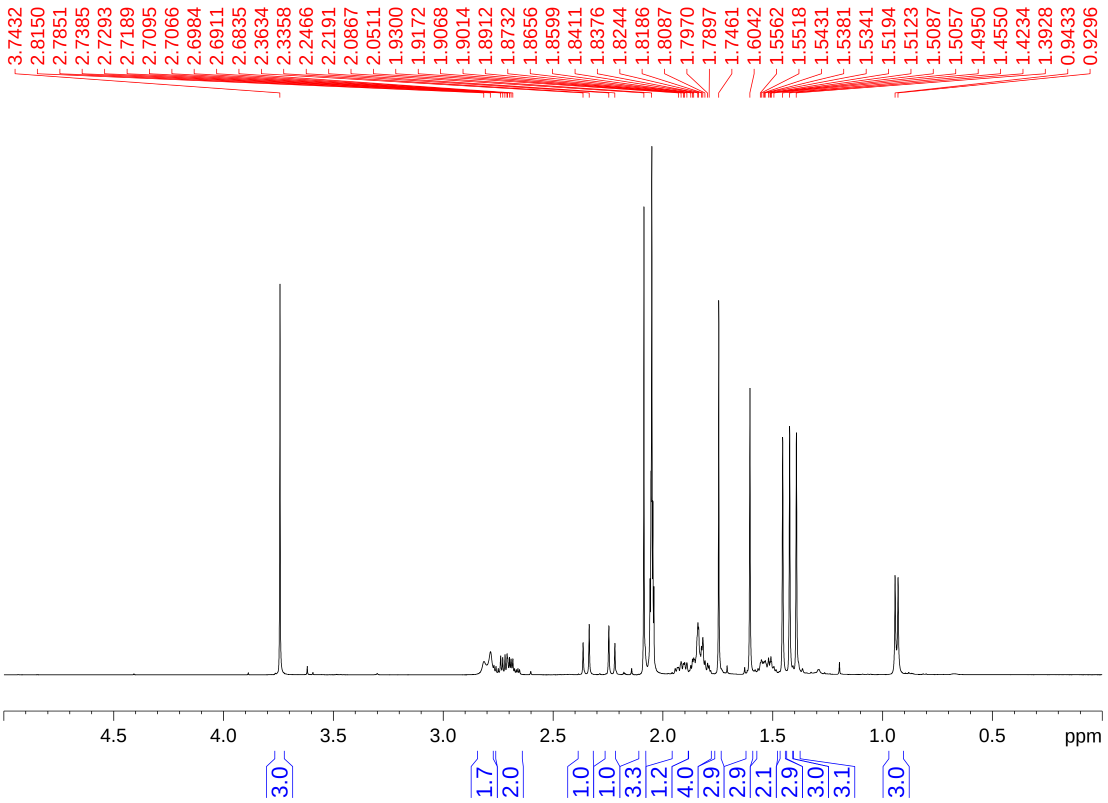


**Figure S16.** $^{1}$H NMR spectrum (500 MHz, Acetone-$d_6$) of **3**

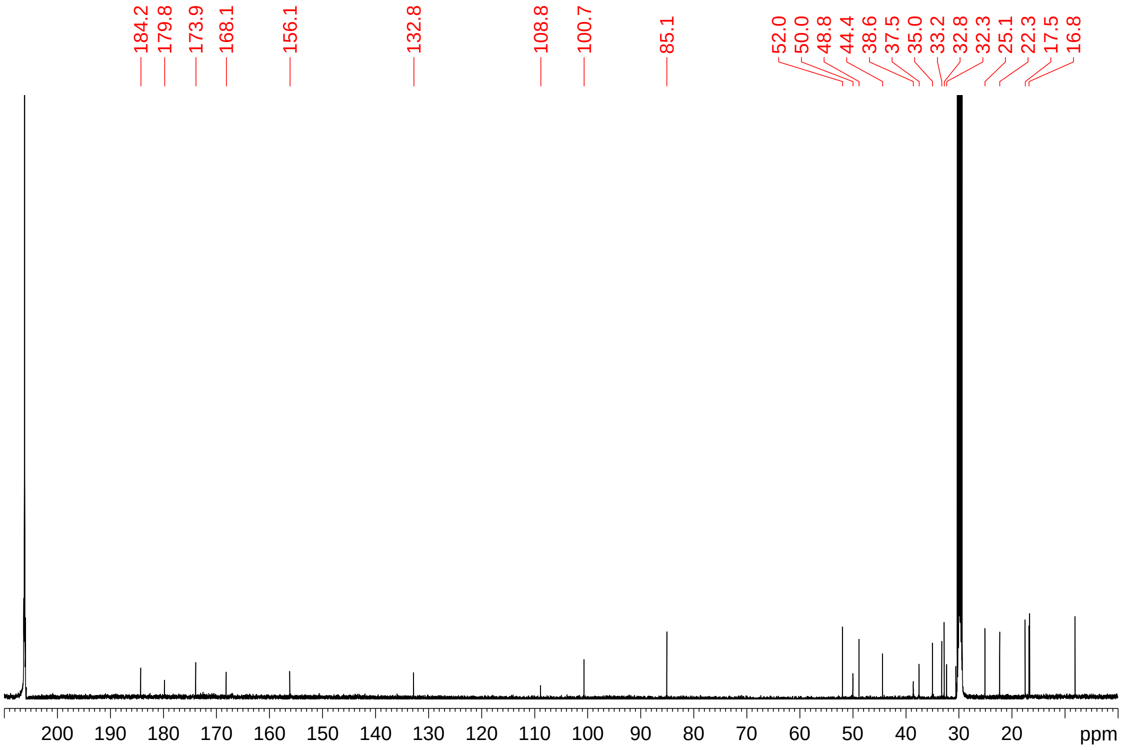


**Figure S17.** $^{13}$C NMR spectrum (125 MHz, Acetone-$d_6$) of **3**

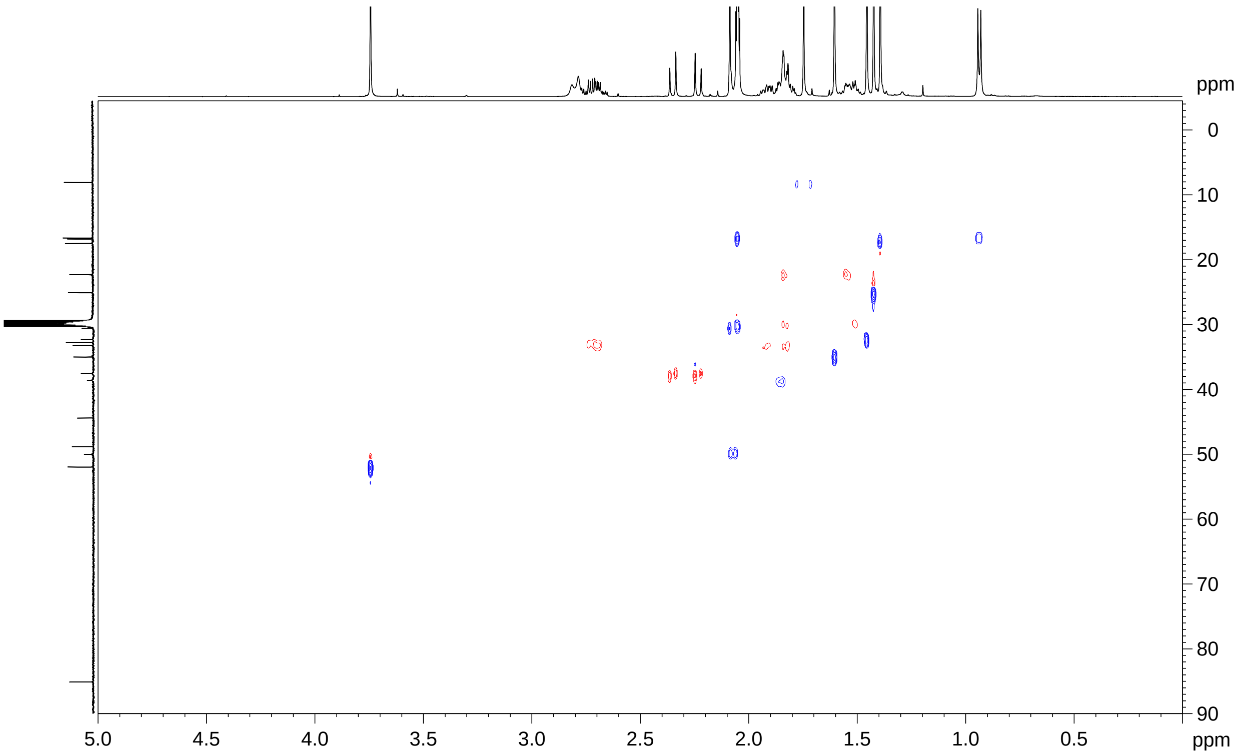


**Figure S18.** HSQC spectrum (500 MHz, Acetone-$d_6$) of **3**

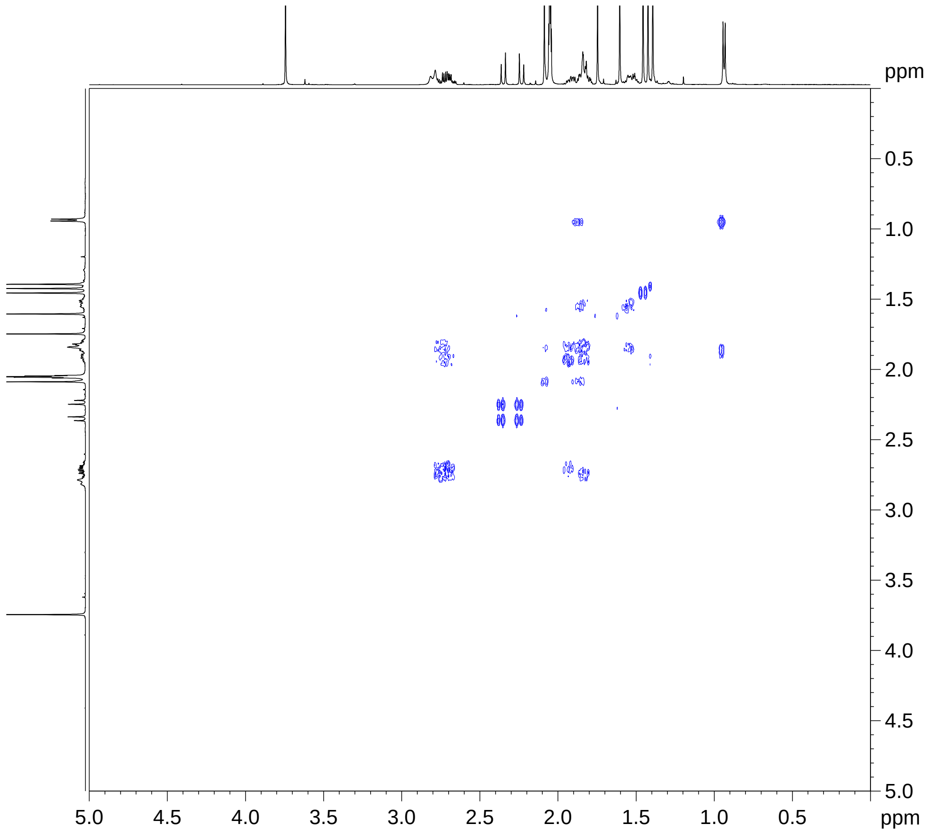


**Figure S19.** $^1H$ -$^1H$ COSY spectrum (500 MHz, Acetone-$d_6$) of **3**

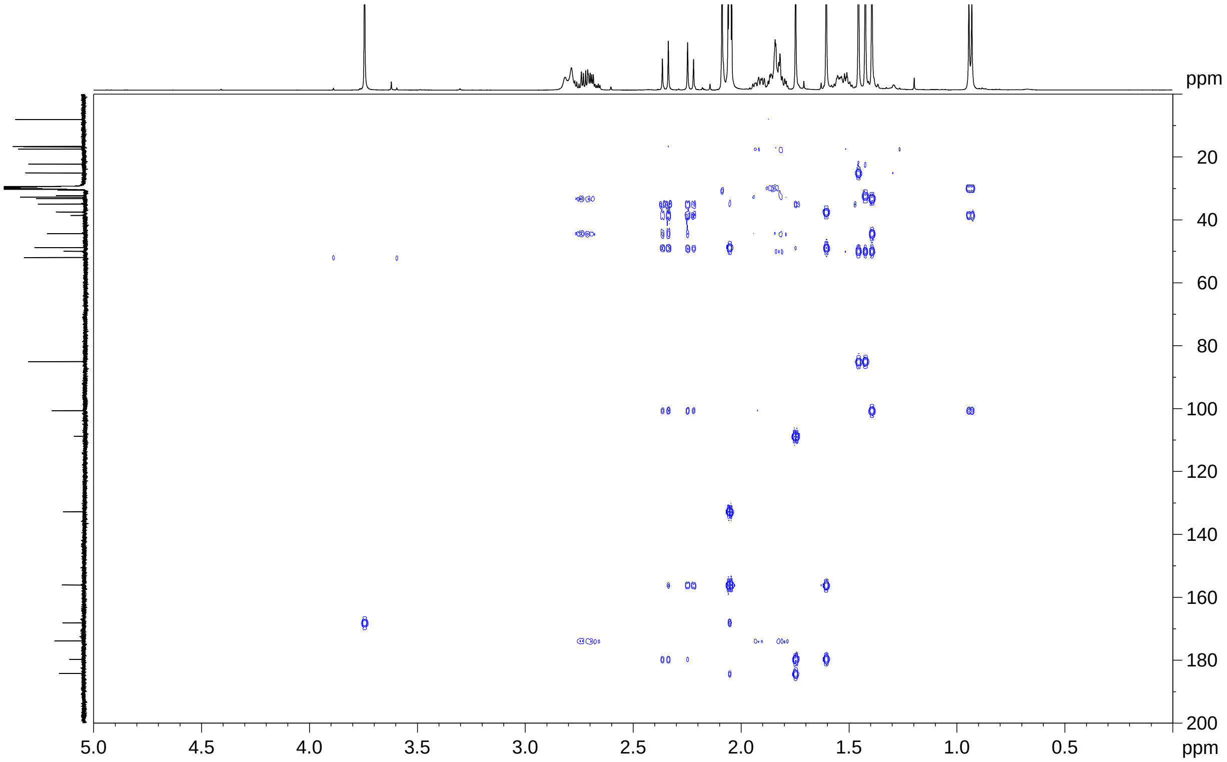


**Figure S20.** HMBC spectrum (500 MHz, Acetone-$d_6$) of **3**

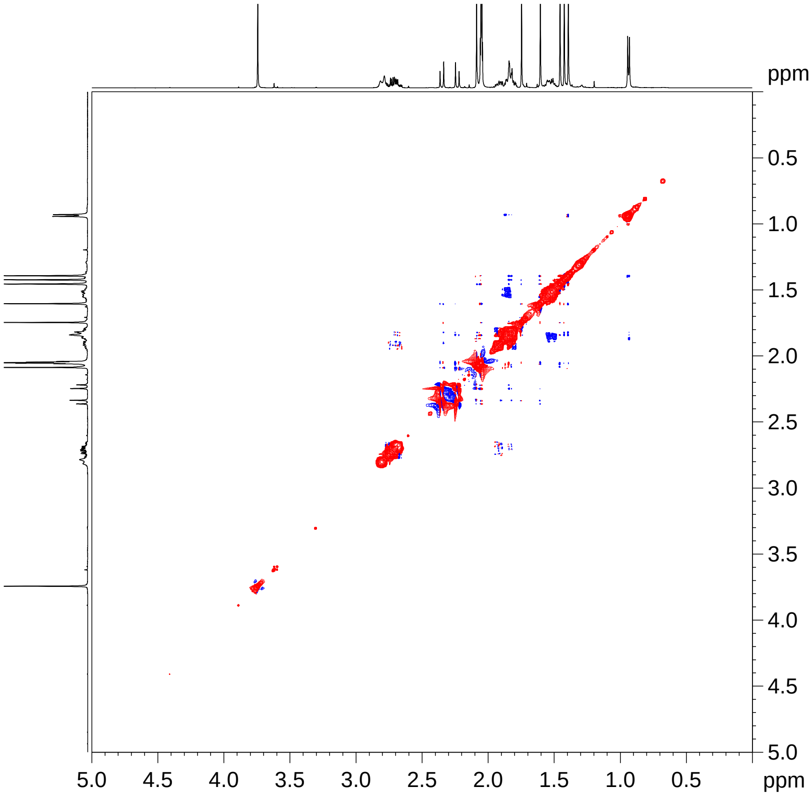


**Figure S21.** ROESY spectrum (500 MHz, Acetone-$d_6$) of **3**

Table S1. $^{13}C$ NMR data for compound **4**

| Position | Compound **4** (Acetone-$d_6$) | Asnovolin I ($CDCl_3$) |
|---|---|---|
| | $\delta_C$ | $\delta_C$ |
| 1 | 34.8, $CH_2$ | 34.4, $CH_2$ |
| 2 | 29.8, $CH_2$ | 29.1, $CH_2$ |
| 3 | 174.6, C | 174.2, C |
| 4 | 75.0, C | 75.0, C |
| 5 | 49.7, CH | 48.1, CH |
| 6 | 23.5, $CH_2$ | 23.0, $CH_2$ |
| 7 | 29.3, $CH_2$ | 28.8, $CH_2$ |
| 8 | 39.6, CH | 39.2, CH |
| 9 | 101.2, C | 99.8, C |
| 10 | 45.6, C | 45.0, C |
| 11 | 37.1, $CH_2$ | 37.1, $CH_2$ |
| 12 | 48.9, C | 48.1, C |
| 13 | 155.9, C | 155.2, C |
| 14 | 133.1, C | 132.4, C |
| 15 | 184.2, C | 184.2, C |
| 16 | 106.9, C | 107.9, C |
| 17 | 181.7, C | 179.6, C |
| 18 | 35.2, $CH_3$ | 35.0, $CH_3$ |
| 19 | 16.7, $CH_3$ | 16.9, $CH_3$ |
| 20 | 168.1, C | 167.6, C |
| 21 | 52.0, $OCH_3$ | 52.4, $CH_3$ |
| 22 | 8.3, $CH_3$ | 8.3, $CH_3$ |
| 23 | 21.3, $CH_3$ | 22.8, $CH_3$ |
| 24 | 16.7, $CH_3$ | 16.3, $CH_3$ |
| 25 | 30.6, $CH_3$ | 32.4, $CH_3$ |
| 26 | 34.8, $CH_3$ | 32.8, $CH_3$ |
| 27 | 51.7, $OCH_3$ | 52.0, $OCH_3$ |

Table S2. $^{13}$C NMR data for compound **5**

| Position | Compound **5** (Acetone-$d_6$) | (2'*E*,4'*E*,6'*E*)-6-(1'-carboxyocta-2',4',6'-triene)-9-hydroxydrim-7-ene-11,12-olide (DMSO-$d_6$) |
|---|---|---|
| | $\delta_C$ | $\delta_C$ |
| 1 | 30.7, $CH_2$ | 29.4, $CH_2$ |
| 2 | 18.6, $CH_2$ | 17.3, $CH_2$ |
| 3 | 45.6, $CH_2$ | 44.3, $CH_2$ |
| 4 | 32.8, C | 33.2, C |
| 5 | 45.7, CH | 44.1, CH |
| 6 | 66.9, CH | 65.7, CH |
| 7 | 123.1, CH | 121.3, CH |
| 8 | 136.1, C | 136.5, C |
| 9 | 74.7, C | 73.0, C |
| 10 | 38.6, C | 37.2, C |
| 11 | 174.9, C | 174.2, C |
| 12 | 69.0, $CH_2$ | 68.1, $CH_2$ |
| 13 | 34.4, $CH_3$ | 32.0, $CH_3$ |
| 14 | 25.1, $CH_3$ | 24.1, $CH_3$ |
| 15 | 19.0, $CH_3$ | 18.2, $CH_3$ |
| 1' | 166.4, C | 165.2, C |
| 2' | 120.9, CH | 119.5, CH |
| 3' | 146.4, CH | 145.4, CH |
| 4' | 128.5, CH | 127.3, CH |
| 5' | 142.6, CH | 141.7, CH |
| 6' | 132.2, CH | 131.3, CH |
| 7' | 137.6, CH | 135.5, CH |
| 8' | 18.6, $CH_3$ | 18.2, $CH_3$ |

Table S3. $^{13}$C NMR data for compound **6**

| Positi on | Compound **6** (Acetone-$d_6$) | (2'*E*,4'*E*,6'*E*)-6-(1'-carboxyocta-2',4',6'-triene)-11,12-epoxy-9,11-dihydroxydrim-7-ene (DMSO-$d_6$) |
|---|---|---|
| | $\delta_C$ | $\delta_C$ |
| 1 | 33.2, $CH_2$ | 33.3, $CH_2$ |
| 2 | 19.1, $CH_2$ | 19.4, $CH_2$ |
| 3 | 45.6, $CH_2$ | 32.8, C |
| 4 | 32.8, C | 32.8, C |
| 5 | 45.7, CH | 45.7, CH |
| 6 | 67.5, CH | 68.7, CH |
| 7 | 121.3, CH | 121.2, CH |
| 8 | 143.2, C | 143.9, C |
| 9 | 78.0, C | 78.5, C |
| 10 | 39.1, C | 39.9, C |
| 11 | 98.5, CH | 99.5, CH |
| 12 | 67.1, $CH_2$ | 67.9, $CH_2$ |
| 13 | 32.6, $CH_3$ | 33.6, $CH_3$ |
| 14 | 25.0, $CH_3$ | 25.5, $CH_3$ |
| 15 | 18.8, $CH_3$ | 19.7, $CH_3$ |
| 1' | 166.5, C | 168.4, C |
| 2' | 119.0, CH | 120.2, CH |
| 3' | 146.0, CH | 147.3, CH |
| 4' | 128.6, CH | 128.9, CH |
| 5' | 142.4, CH | 143.4, CH |
| 6' | 132.3, CH | 132.8, CH |
| 7' | 135.9, CH | 136.8, CH |
| 8' | 18.6, $CH_3$ | 18.9, $CH_3$ |

Table S4. $^{13}C$ NMR data for compound **7**

| Position | Compound **7** ($CDCl_3$) | cinereain ($CDCl_3$) |
|---|---|---|
| | $\delta_C$ | $\delta_C$ |
| 1 | 60.7, CH | 60.7, CH |
| 2 | 163.0, C | 162.9, C |
| 4 | 124.8, C | 124.6, C |
| 5 | 161.0, C | 160.9, C |
| 7 | 164.6, C | 165.1, C |
| 9 | 143.2, CH | 143.1, CH |
| 10 | 117.3, CH | 117.2, CH |
| 11 | 127.8, CH | 127.6, CH |
| 12 | 125.8, CH | 125.7, CH |
| 13 | 110.6, C | 110.3, C |
| 14 | 149.6, C | 149.6, C |
| 16 | 33.6, CH | 33.4, CH |
| 17 | 22.1, $CH_3$ | 22.1, $CH_3$ |
| 18 | 17.1, $CH_3$ | 17.0, $CH_3$ |
| 20 | 128.7, C | 129.2, C |
| 21 | 26.4, CH | 26.2, CH |
| 22 | 22.2, $CH_3$ | 22.1, $CH_3$ |
| 23 | 19.9, $CH_3$ | 19.7, $CH_3$ |

Table S5. $^{13}$C NMR data for compound **8**

| Position | Compound **8** (Acetone-$d_6$) | carnequinazoline A ($CDCl_3$) |
|---|---|---|
| | $\delta_C$ | $\delta_C$ |
| 1 | 164.6, C | 164.6, C |
| 3 | 126.0, C | 125.6, C |
| 4 | 146.6, C | 144.9, C |
| 6 | 148.1, C | 147.2, C |
| 7 | 128.0, CH | 127.5, CH |
| 8 | 135.1, CH | 134.7, CH |
| 9 | 127.1, CH | 126.9, CH |
| 10 | 127.1, CH | 127.0, CH |
| 11 | 120.8, C | 120.2, C |
| 12 | 160.9, C | 160.8, C |
| 14 | 60.8, CH | 60.3, CH |
| 15 | 33.6, CH | 33.5, CH |
| 16 | 17.8, $CH_3$ | 17.5, $CH_3$ |
| 17 | 19.7, $CH_3$ | 19.7, $CH_3$ |
| 18 | 126.1, CH | 125.5, CH |
| 19 | 26.1, CH | 26.3, CH |
| 20 | 22.3, $CH_3$ | 22.2, $CH_3$ |
| 21 | 22.1, $CH_3$ | 22.1, $CH_3$ |

Table S6. $^{13}C$ NMR data for compound **9**

| Position | Compound **9** ($CDCl_3$) | carnequinazoline B ($CDCl_3$) |
|---|---|---|
| | $\delta_C$ | $\delta_C$ |
| 1 | 165.3, C | 165.0, C |
| 3 | 125.4, C | 125.4, C |
| 4 | 144.6, C | 144.4, C |
| 6 | 135.7, C | 135.6, C |
| 7 | 151.5, C | 151.4, C |
| 8 | 117.7, CH | 117.3, CH |
| 9 | 128.1, CH | 127.9, CH |
| 10 | 117.6, CH | 117.5, CH |
| 11 | 120.2, C | 120.1, C |
| 12 | 160.5, C | 160.3, C |
| 14 | 60.5, CH | 60.4, CH |
| 15 | 33.7, CH | 33.5, CH |
| 16 | 17.6, $CH_3$ | 17.5, $CH_3$ |
| 17 | 19.9, $CH_3$ | 19.7, $CH_3$ |
| 18 | 126.6, CH | 126.1, CH |
| 19 | 26.3, CH | 26.2, CH |
| 20 | 22.4, $CH_3$ | 22.2, $CH_3$ |
| 21 | 22.3, $CH_3$ | 22.1, $CH_3$ |

Table S7. $^{13}$C NMR data for compound **10**

| Position | Compound **10** ($CDCl_3$) | carnemycin B ($CD_3OD$) |
|---|---|---|
| | $\delta_C$ | $\delta_C$ |
| 1 | 159.2, C | 164.8, C |
| 2 | 110.4, C | 113.1, C |
| 3 | 159.4, C | 163.0, C |
| 4 | 109.7, CH | 106.3, CH |
| 5 | 142.9, C | 148.4, C |
| 6 | 108.0, C | 111.3, C |
| 7 | 170.3, C | 173.4, C |
| 8 | 34.7, $CH_2$ | 36.4, $CH_2$ |
| 9 | 33.9, $CH_2$ | 36.3, $CH_2$ |
| 10 | 131.2, CH | 132.8, CH |
| 11 | 130.7, CH | 132.7, CH |
| 12 | 130.4, CH | 132.5, CH |
| 13 | 132.3, CH | 134.0, CH |
| 14 | 34.1, $CH_2$ | 36.4, $CH_2$ |
| 15 | 22.0, $CH_2$ | 24.3, $CH_2$ |
| 16 | 13.6, $CH_3$ | 14.6, $CH_3$ |
| 17 | 51.9, $CH_3$ | 53.0, $CH_3$ |
| 1' | 74.2, CH | 76.3, CH |
| 2' | 71.3, CH | 73.7, CH |
| 3' | 78.6, CH | 80.7, CH |
| 4' | 69.9, CH | 72.3, CH |
| 5' | 81.3, CH | 83.2, CH |
| 6' | 60.8, $CH_2$ | 63.3, $CH_2$ |

Table S7. $^{13}$C NMR data for compound **11**

| Position | Compound **11** ($CDCl_3$) | stromemycin (DMSO-$d_6$) |
|---|---|---|
| | $\delta_C$ | $\delta_C$ |
| 1 | 164.2, C | 158.29, C |
| 2 | 112.6, C | 110.56, C |
| 3 | 164.4, C | 159.32, C |
| 4 | 111.0, CH | 109.58, CH |
| 5 | 148.0, C | 142.46, C |
| 6 | 109.3, C | 108.83, C |
| 7 | 170.6, C | 167.36, C |
| 8 | 37.7, $CH_2$ | 34.27, $CH_2$ |
| 9 | 36.2, $CH_2$ | 34.02, $CH_2$ |
| 10 | 131.9, CH | 130.79, CH |
| 11 | 131.9, CH | 130.72, CH |
| 12 | 131.6, CH | 130.24, CH |
| 13 | 133.3, CH | 132.22, CH |
| 14 | 36.9, $CH_2$ | 34.02, $CH_2$ |
| 15 | 23.6, $CH_2$ | 21.96, $CH_2$ |
| 16 | 14.0, $CH_3$ | 13.50, $CH_3$ |
| 17 | 76.1, CH | 74.40, CH |
| 18 | 73.1, CH | 71.53, CH |
| 19 | 80.0, CH | 78.38, CH |
| 20 | 71.6, CH | 69.65, CH |
| 21 | 82.6, CH | 81.21, CH |
| 22 | 62.6, $CH_2$ | 60.51, $CH_2$ |
| 1' | 163.0, C | 157.17, C |
| 2' | 105.8, CH | 107.31, CH |
| 3' | 154.8, C | 151.55, C |
| 4' | 113.3, CH | 113.18, CH |
| 5' | 147.8, C | 142.25, C |
| 6' | 116.4, C | 118.40, C |
| 7' | 173.8, C | 169.73, C |
| 8' | 35.8, $CH_2$ | 33.44, $CH_2$ |
| 9' | 35.8, $CH_2$ | 33.61, $CH_2$ |
| 10' | 132.4, CH | 130.79, CH |
| 11' | 132.6, CH | 130.79, CH |
| 12' | 131.7, CH | 130.31, CH |
| 13' | 133.7, CH | 132.38, CH |
| 14' | 37.7, $CH_2$ | 34.02, $CH_2$ |
| 15' | 23.7, $CH_2$ | 21.97, $CH_2$ |
| 16' | 14.0, $CH_3$ | 13.50, $CH_3$ |

### Preparation of tested compounds and positive controls

Compounds were dissolved in dimethyl sulfoxide (DMSO) with final concentrations ranging from 4,000 to 15.625 µg/mL using a 2-fold serial dilution method. Amphotericin B, ciprofloxacin, or vancomycin dissolved in DMSO with final concentrations of 160, 80, 40, 20, 10, 5, 2.5, 1.25 µg/mL using a 2-fold serial dilution method.

Positive controls were added by a column respectively. while the tested compounds were diluted 2-fold by row in a 96-well microtiter plate.

### Antibacterial assay

The MICs against *S. aureus* and *E. coli* were determined according to the CLSI broth microdilution method and our previous report. *S. aureus* or *E. coli* culture was grown at 37°C with shaking in MHB broth to midlogarithmic phase [optical density at 600 nm = 0.3] and diluted 10 time by MHB broth to give a final concentration of approximately $1 \times 10^6$ CFU/ml. Twofold serial dilutions of the test compounds were prepared in a 96-well microtiter plate by row containing 78 μL of MHB media and 2 μL diluted compounds (The final concentrations were 100, 50, 25, 12.5, 6.25, 3.125, 1.5625, 0.78125, 0.390625 µg/mL, respectively.), while the positive controls were diluted 2-fold by row in a 96-well microtiter plate (The final concentrations were 4, 2, 1, 0.5, 0.25, 0.125, 0.0625, 0.03125, 0.015625 µg/mL, respectively). The MIC was defined as the lowest concentrations of samples with no visible growth of bacteria after incubation at 37°C for 18 hours. All MIC assays were performed in two independent experiments.

### Antifungal assay

MICs against *C. albicans* were determined according to the CLSI guideline and our previous report. *C. albicans* culture was grown at 30°C with shaking in RPMI 1640 medium to midlogarithmic phase [optical density at 530 nm = 0.5] and diluted 500 time by RPMI 1640 medium to give a final concentration of approximately $1 \times 10^4$ CFU/ml. Twofold serial dilutions of the test compounds were prepared in a 96-well microtiter plate by row containing 78 μL of RPMI 1640 media and 2 μL diluted compounds (The final concentrations were 100, 50, 25, 12.5, 6.25, 3.125, 1.5625, 0.78125, 0.390625 µg/mL, respectively.), while the positive control was diluted 2-fold by column in a 96-well microtiter plate (The final concentrations were 4, 2, 1, 0.5, 0.25, 0.125, 0.0625 µg/mL, respectively). The MIC was defined as the lowest concentrations of samples with no visible growth of fungus after incubation at 35°C for 24 hours. All MIC assays were performed in two independent experiments.
